# Multimodal Sensor Fusion and Temporal Deep Learning for Computer Numerical Control Toolpath and Condition Classification: A Cross-Validated Ablation Study

**DOI:** 10.3390/s26082405

**Published:** 2026-04-14

**Authors:** Stephen S. Eacuello, Romesh S. Prasad, Manbir S. Sodhi

**Affiliations:** Department of Industrial Systems Engineering, University of Rhode Island, Kingston, RI 02881, USA; seacuello@uri.edu (S.S.E.); romeshsatish.prasad@uri.edu (R.S.P.)

**Keywords:** computer numerical control machining, multimodal sensor fusion, sensor modality selection, deep learning, temporal modeling, feature ablation, cross-validation, process monitoring, denoising autoencoder

## Abstract

Classifying which operation a Computer Numerical Control (CNC) machine is executing, not just detecting whether it is functioning correctly, is a monitoring challenge that existing sensor-based studies rarely address. Unlike tool wear estimation, operation-type classification must resolve toolpath strategies and cutting conditions within heterogeneous, noisy sensor streams in which modalities differ widely in their discriminative value. Which sensors are genuinely necessary, and how many can be removed before performance degrades, directly informs retrofit cost and monitoring system design. We present a systematic cross-validated ablation study for a nine-class CNC toolpath and condition classification task, using 120 operation files collected from a desktop CNC mill instrumented with six distributed sensor units spanning inertial, acoustic, environmental, and electrical modalities. To handle multimodal fusion under sensor noise, we introduce the Multimodal Denoising Temporal Attention Encoder with Long Short-Term Memory (MM-DTAE-LSTM), which combines learned modality weighting, cross-modal attention, and a self-supervised denoising objective, followed by recurrent temporal modeling for classification. We evaluate MM-DTAE-LSTM against five baseline model families across five cumulative sensor-ablation levels and ten temporal resolutions, using file-level cross-validation to prevent data leakage from overlapping windows. MM-DTAE-LSTM maintains 92.5% classification accuracy when nearly half the sensor channels are removed (56 of 110 features), whereas simpler baselines degrade by up to 10.7 percentage points under the same reduction. Analysis of variance reveals that pressure channels encode session-level atmospheric variation rather than machining dynamics, exposing how models that cannot suppress uninformative modalities rely on environmental confounds rather than machining physics. Together, these findings translate into concrete sensor-selection and deployment recommendations for cost-effective CNC process monitoring at under USD 500 in hardware, though generalization to industrial machines, diverse materials, and production environments requires further validation.

## 1. Introduction

Computer Numerical Control (CNC) machines execute precise, multi-axis toolpaths that underpin manufacturing across aerospace, automotive, medical device, and defense sectors. A single undetected deviation in spindle speed, feed rate, or depth of cut can scrap parts, damage tooling, or cause costly unplanned downtime [1]. Sensor-based monitoring offers a path toward continuous process verification. It confirms that what the machine physically executed matches what was commanded and lays the groundwork for anomaly detection in increasingly networked manufacturing environments [2,3].

Most prior work in CNC sensor monitoring addresses tool wear estimation and binary fault detection using a small number of sensors, typically two to eight channels [1,4,5]. Operation-type classification has received far less attention. This is a fundamentally different problem from tool wear estimation. Rather than determining how worn the tool is, operation-type classification determines what machining strategy is currently being executed. Verifying that an intended operation occurred requires recognizing the toolpath strategy and cutting condition, not just flagging anomalous vibration. Systematic evidence about which sensor modalities contribute to such classification, and how performance degrades as sensors are removed, is largely absent from the literature.

The closest analogous work uses distributed sensors to classify operation type from physical signals in additive manufacturing [6,7,8]. In processes such as Fused Deposition Modeling, material is deposited along a single axis in a controlled environment. Sensors can be placed freely without risk of damage from cutting forces or chip ejection. Replicating this approach in CNC milling is substantially harder. The cutting tool engages a rigid workpiece under high forces, generating vibration, acoustic emission, and thermal gradients across all axes simultaneously. These conditions corrupt sensor signals, introduce structured noise, and physically risk sensors mounted near the cutting zone. The machine’s structural response is also spatially non-uniform. A spindle-mounted sensor captures different dynamics than one on the frame or bed. Distributed sensors across the machine structure capture complementary information, but identifying the minimum set required for reliable classification is a design challenge that additive manufacturing studies have not had to confront.

We address this challenge through a systematic cross-validated ablation study on nine-class CNC toolpath and condition classification. Three aspects distinguish this study from prior work. The sensor ablation is cumulative and model-comparative, quantifying degradation across five baseline model families rather than a single model. The evaluation uses file-level cross-validation to prevent data leakage from overlapping windows. A confound analysis identifies which channels carry genuine machining signal versus session-level environmental variation. Together, these design choices produce actionable sensor-selection guidance grounded in both classification performance and physical interpretability.

This study is guided by four research questions. First, which sensor modalities carry genuine discriminative signal for CNC operation-type classification, and which encode environmental confounds? Second, how much does classification performance degrade as sensors are progressively removed, and does a complex temporal encoder degrade more or less than simpler baselines? Third, which architectural components of a multimodal temporal encoder contribute most to its performance on this task? Fourth, what is the minimum sensor configuration that preserves practical classification accuracy for cost-effective deployment? These questions are answered in Section 4 and Section 5 and summarized in Section 6.

This paper makes the following contributions:

1. **Systematic sensor ablation for deployment guidance.** A five-level cumulative modality ablation that progressively removes low-value and confound-prone channels, quantifying the minimum sensor configuration that preserves strong classification performance.
2. **MM-DTAE-LSTM architecture and component evaluation.** A multimodal encoder combining learned modality weighting, cross-modal attention, and a self-supervised denoising objective, followed by recurrent temporal modeling for classification. An architectural and capacity ablation (Section 4.6) identifies the denoising objective as the critical component, accounting for 7.9 macro-F1 points, while cross-modal attention and model capacity contribute minimally at this data scale.
3. **Leakage-resistant evaluation and baseline benchmarking.** Five-fold file-level stratified cross-validation across 120 operation files, with MM-DTAE-LSTM compared against five baseline model families under identical protocols, feature configurations, and temporal resolutions.
4. **Confound analysis.** One-way analysis of variance demonstrating that pressure channels encode session-level environmental variation rather than machining dynamics, with implications for sensor selection and evaluation methodology.

The remainder of this paper is organized as follows. Section 2 reviews related work. Section 3 describes the experimental setup and methods. Section 4 presents results. Section 5 discusses implications, limitations, and deployment recommendations.

## 2. Related Work

This section reviews prior work in three areas that directly inform the present study. Section 2.1 surveys CNC process monitoring and multimodal sensor fusion. Section 2.2 covers temporal deep learning for industrial sensor streams. Section 2.3 addresses anomaly detection and positions this work relative to the literature.

### 2.1. CNC Process Monitoring and Multimodal Sensor Fusion

Sensor-based monitoring of machining processes has a well-established foundation. Early work identified force, vibration, acoustic emission, temperature, and motor current as complementary sensing modalities [1]. Hand-crafted features extracted via Fourier and wavelet transforms became standard practice for tool condition monitoring [5]. Deep learning subsequently enabled end-to-end feature learning from raw signals. This improved robustness to noise and reduced reliance on domain-specific feature engineering [9,10]. Convolutional neural networks applied to vibration spectrograms and raw multi-channel signals have shown strong results on bearing fault detection and surface roughness classification [11,12,13].

Multimodal fusion strategies span a spectrum from early fusion to late fusion. Early fusion concatenates features from all modalities before learning. Late fusion combines independently trained classifiers. Intermediate approaches using cross-modal attention have shown particular promise when sensor modalities are complementary rather than redundant [14,15,16]. Wang et al. [17] demonstrated that coupling a denoising Transformer autoencoder with a downstream classifier improves robustness under sensor noise in multi-sensor tool wear classification. This directly motivates the denoising component in our architecture. A comprehensive review of multi-sensor fusion for CNC monitoring by Tsanousa et al. [18] confirms that the field remains dominated by tool condition tasks.

Despite this progress, two gaps persist. Nearly all prior work frames the problem as tool wear estimation or binary fault detection. Operation-type classification, distinguishing what machining strategy is being executed rather than how worn the tool is, has received little attention. When multiple sensors are used, systematic evidence about which modalities are necessary is rare. Ablation studies that progressively remove sensor groups to quantify their marginal contribution [19,20] are essentially absent from the CNC monitoring literature. Practitioners are left without quantitative guidance on sensor selection.

### 2.2. Temporal Deep Learning for Industrial Sensor Streams

Machining sensor data are inherently sequential. Toolpath geometry unfolds over time, producing direction-change transients, step-over periodicity, and plunge-clear alternation patterns that carry classification-relevant information. Long Short-Term Memory networks [21] remain widely used for such continuous sequential data. They capture long-range temporal dependencies that windowed feature approaches discard. Encoder–decoder architectures with attention mechanisms [22,23] extend sequence modeling to variable-length outputs. The Transformer architecture [24] demonstrates strong performance on time-series benchmarks [25]. For lower-dimensional industrial sensor streams with continuous dynamics, recurrent networks often retain practical advantages over attention-only models. This is especially true when causal ordering matters for streaming inference [26].

Denoising autoencoders [27] learn representations that are robust to input corruption. They train to reconstruct clean inputs from noisy ones. Applied to industrial sensor data, this objective encourages the encoder to extract signal structure rather than fitting noise. This is especially valuable when sensors are subject to mechanical vibration, electromagnetic interference, and thermal drift, all of which are characteristic of CNC milling environments. These considerations motivate our architectural combination of cross-modal attention for modality fusion, a Long Short-Term Memory for temporal modeling, and a self-supervised denoising objective on the encoder.

### 2.3. Anomaly Detection and Study Positioning

Coelho et al. [28] applied one-dimensional convolutional neural networks to current and vibration signals for CNC anomaly detection. Their approach achieved strong binary accuracy but did not distinguish between operation types. Kimmell et al. [29] demonstrated sensor-based detection of control injection attacks in CNC machining using energy anomaly thresholds. A substantial body of related work addresses process verification in additive manufacturing. Side-channel signals from Fused Deposition Modeling printers have been used to infer G-code geometry with high fidelity across acoustic, magnetic, and video modalities [6,7,8,30,31]. CNC milling presents a considerably harder signal environment. Simultaneous multi-axis interpolation, nonlinear force-engagement relationships, and structural vibration through the machine frame produce sensor signatures with far greater complexity than single-axis filament deposition.

Table 1 positions our work against recent experimental studies identified through searches of IEEE Xplore, Scopus, and Google Scholar using the terms “CNC monitoring,” “machining sensor classification,” “multimodal sensor fusion,” and “tool condition monitoring deep learning,” filtered to studies published between 2016 and 2025 that report classification results on experimental data. Studies were selected to represent the range of tasks, sensor counts, and validation strategies present in the field. No prior study combines operation-type classification, multimodal sensor ablation, and file-level cross-validation together.

## 3. Materials and Methods

**Figure 1:**
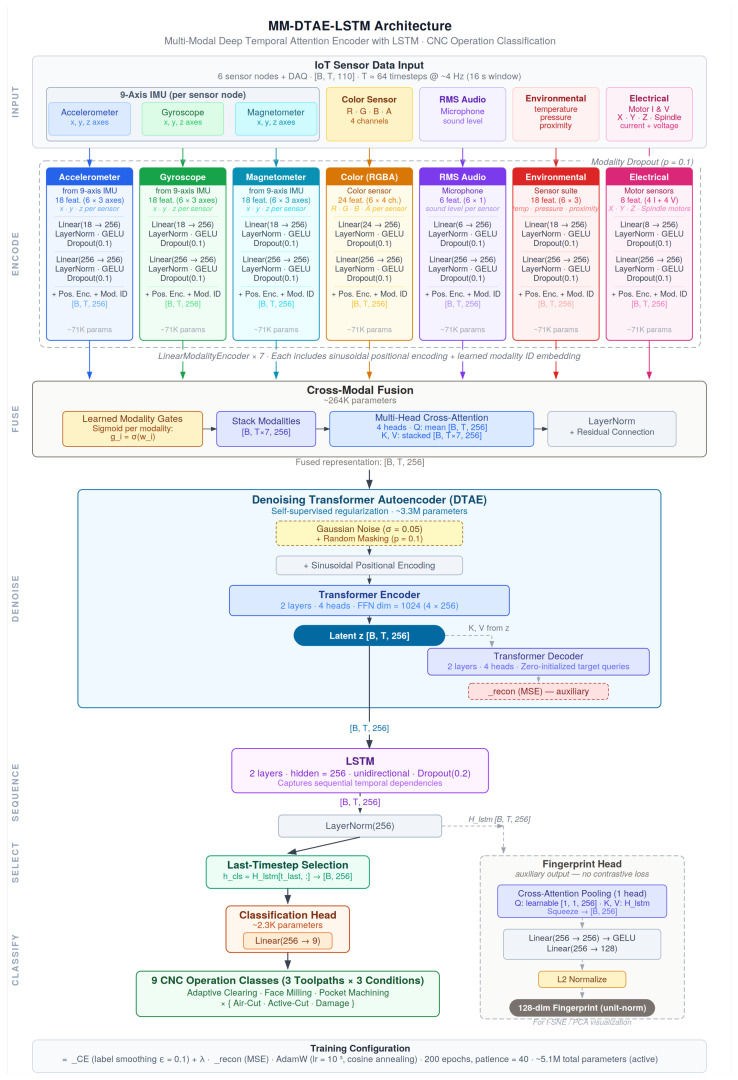
MM-DTAE-LSTM architecture. Modality-specific projections feed gated cross-modal attention, a denoising transformer autoencoder, and a unidirectional Long Short-Term Memory network for 9-class classification.

**Figure 2:**
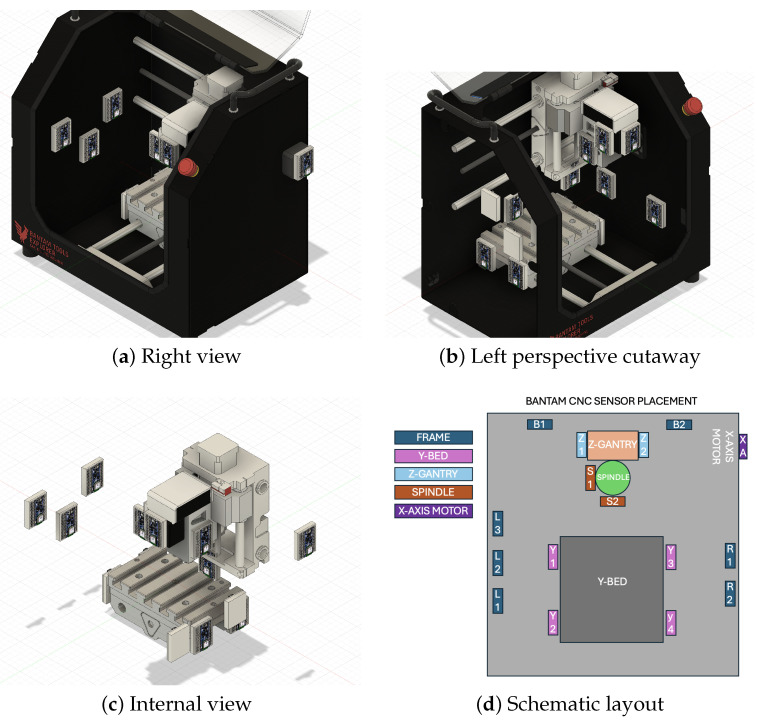
Sensor placement on the Bantam Tools Explorer CNC mill. (**a**) Right view showing frame-mounted sensors. (**b**) Left perspective cutaway revealing internal sensor positions. (**c**) Internal view of spindle and bed sensors. (**d**) Schematic layout of all 16 sensor positions. Arduino Nano 33 BLE Sense Lite boards are mounted on breadboards affixed with cyanoacrylate adhesive for rigid structural coupling. Of the 16 deployed sensors, 6 maintain ≥93% data consistency and are used for cross-validated experiments (Table A1).

**Figure 3:**
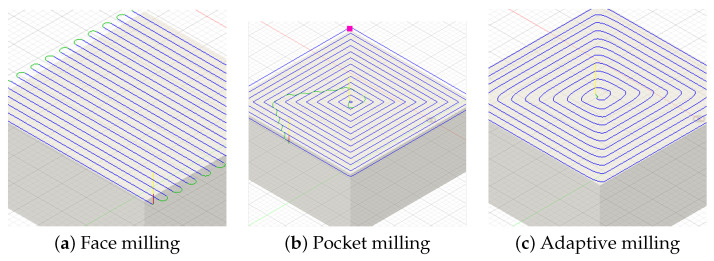
Computer-aided manufacturing toolpath strategies used in this study: (**a**) face milling sweeps in parallel linear passes with step-over transitions; (**b**) pocket milling alternates between axial plunges and lateral clearing passes; (**c**) adaptive (trochoidal) milling follows curved, constant-engagement paths with rapid direction reversals.

This section describes the experimental design, model architecture (Figure 1), sensor instrumentation (Figure 2), and evaluation methodology. Section 3.1 defines the classification task and the three toolpath strategies (Figure 3). Section 3.2 and Section 3.3 describe the MM-DTAE-LSTM architecture and training configuration. Section 3.4, Section 3.5 and Section 3.6 cover the experimental setup, modality structure, and baseline models. Section 3.7, Section 3.8 and Section 3.9 define the evaluation protocol and ablation designs.

### 3.1. Classification Task

The classification task spans nine classes defined by the intersection of three toolpath strategies and three operating conditions (Table 2). This structure captures two independent sources of variation. Toolpath strategy determines the geometric trajectory of the cutting tool. Operating condition determines whether the machine is engaging material, running dry, or operating with a degraded spindle.

The three toolpath strategies shown in Figure 3 produce geometrically and dynamically distinct motion profiles.

- **Adaptive Clearing** follows trochoidal paths that maintain near-constant radial engagement. Frequent direction reversals and high-jerk cornering at each arc produce periodic acceleration transients.
- **Face Milling** executes parallel linear passes with periodic step-over transitions. The result is quasi-steady gantry motion punctuated by rapid repositioning at each pass boundary.
- **Pocket Machining** clears enclosed regions through alternating axial plunge entries and lateral offset passes. This produces bimodal motion signatures with distinct axial and lateral frequency content.

The three operating conditions span normal and degraded spindle states. Air-cut operations run the spindle with no material engagement. Active-cut operations engage a workpiece at a fixed depth of cut. Damage operations introduce deliberate spindle degradation during air-cutting. Together with the three toolpath strategies, these conditions yield the nine classification targets in Table 2.

### 3.2. The MM-DTAE-LSTM Architecture

The overall architecture is shown in Figure 1 and described below.

#### 3.2.1. Design Rationale

The architecture is shaped by three properties of CNC multimodal sensor data that make naive fusion approaches insufficient. First, sensor modalities are physically heterogeneous. Accelerometers measure structural acceleration in metres per second squared; gyroscopes measure angular rate; barometric sensors measure atmospheric pressure; microphones capture acoustic emission amplitude. These signals differ not only in physical units but also in dynamic range, noise floor, and temporal structure. Concatenating raw channels from all modalities into a single input vector and passing them through a shared encoder forces the model to resolve these differences implicitly, making learning harder and representations less interpretable. Separate per-modality encoders address this by first normalizing each modality into a common learned representation space before any fusion occurs.

Second, modalities are not equally informative. Some sensor positions and modality types carry strong discriminative signal for operation classification; others carry weak signal or encode environmental confounds unrelated to machining dynamics. A fusion architecture that weights all modalities equally is pulled toward confound-laden channels. Learned importance gates allow the model to suppress uninformative modalities automatically, with the weight of each modality emerging from training rather than being set by hand.

Third, the data are sequential and the ordering matters. A machining operation unfolds over time: the early portion of a pocket operation—a plunge entry—looks different from its middle portion (lateral clearing), which looks different from its end. This temporal progression is part of the classification signal. A model that collapses all timesteps into a single feature vector before classifying discards this information. The architecture preserves and exploits temporal structure at every stage.

These three considerations motivate the five-stage design: (1) per-modality projection into a shared space, (2) gated cross-modal fusion, (3) denoising representation learning, (4) temporal modeling, and (5) classification. Whether each stage is empirically justified at this data scale is evaluated in Section 4.6.

Algorithm 1 summarizes the forward pass. Each stage is described in the following subsections.
**Algorithm 1** MM-DTAE-LSTM Forward Pass**Require:** Modalities $\left\{M_{1},\ldots ,M_{7}\right\}$, sequence lengths *ℓ***Ensure:** Class prediction $\hat{y}\in R^{9}$1:2: **Stage 1: Modality Projection**3: **for** i=1 to 7 **do**4:      ${\tilde{H}}_{i}\leftarrow {\mathrm{MLP}}_{i}\left(M_{i}\right)+\mathrm{PE}\left(T\right)+e_{i}$5: **end for**6:7: **Stage 2: Cross-Modal Fusion**8: **if** training **then**9:      Apply modality dropout (p=0.1, keep ≥1)10: **end if**11: $g_{i}\leftarrow \sigma \left(w_{i}\right)$ for each modality                                                                       *(learned gates)*12: $H_{\mathrm{fused}}\leftarrow \mathrm{LN}\;\left(\mathrm{LN}\left(\bar{H}+\mathrm{MHA}\left(\bar{H},\left[{\tilde{H}}_{1:7}\right],\left[{\tilde{H}}_{1:7}\right]\right)\right)+\frac{\sum_{i}g_{i}{\tilde{H}}_{i}}{\sum_{i}g_{i}+\epsilon }\right)$13:14: **Stage 3: Denoising Autoencoder**15: **if** training **then**16:      Corrupt: $\tilde{H}\leftarrow H_{\mathrm{fused}}+N\left(0,0.05^{2}\right)$; mask 10%17:      $z\leftarrow \mathrm{TransformerEnc}(\tilde{H})$18:      $\hat{H}\leftarrow \mathrm{TransformerDec}\left(0,z\right)$                                                                  *(reconstruction)*19: **else**20:      $z\leftarrow \mathrm{TransformerEnc}(H_{\mathrm{fused}})$21: **end if**22:23: **Stage 4–5: Temporal Modeling & Classification**24: $H_{\mathrm{lstm}}\leftarrow \mathrm{LN}\left(\mathrm{LSTM}\left(z\right)\right)$25: $h_{\mathrm{cls}}\leftarrow H_{\mathrm{lstm}}\left[l_{b}-1,:\right]$                                                                                     *(last timestep)*26: $\hat{y}\leftarrow \mathrm{softmax}\left(W_{\mathrm{cls}}h_{\mathrm{cls}}+b_{\mathrm{cls}}\right)$27:28: **return** $\hat{y}$; $L_{\mathrm{recon}}=\mathrm{MSE}\left(\hat{H},H_{\mathrm{fused}}\right)$ if training

#### 3.2.2. Stage 1: Modality Projection

Each of the seven modality groups has a different number of input channels, different physical units, and different noise characteristics. Projecting all modalities jointly without modality-specific weights would require a single linear layer to simultaneously resolve, for example, the three-component structure of accelerometer data and the single-channel structure of root mean square audio—two signals with entirely different dynamic ranges and spectral content. Instead, each modality $M_{i}\in R^{T\times C_{i}}$ is independently projected into a shared $d_{\mathrm{model}}$-dimensional space through its own two-layer multilayer perceptron, where each layer applies a linear transform, layer normalization [32], Gaussian error linear unit activation, and dropout [33]:

(1) $$H_{i}^{\left(1\right)}=\mathrm{Drop}\left(\mathrm{GELU}(\mathrm{LN}\left(M_{i}W_{i}^{\left(1\right)}+b_{i}^{\left(1\right)}\right))\right),\;H_{i}=\mathrm{Drop}\left(\mathrm{GELU}(\mathrm{LN}\left(H_{i}^{\left(1\right)}W_{i}^{\left(2\right)}+b_{i}^{\left(2\right)}\right))\right)$$

After projection, sinusoidal positional encodings and a learned modality identity embedding $e_{i}$ are added. The positional encoding preserves temporal ordering within each modality; the identity embedding distinguishes which modality each token came from so that the subsequent cross-modal attention can reason about inter-modality relationships rather than treating all tokens as equivalent:

(2) $${\tilde{H}}_{i}=H_{i}+\mathrm{PE}\left(T\right)+e_{i}$$

At this point, all seven modalities are represented in the same $d_{\mathrm{model}}$-dimensional space and are ready to be compared and combined.

#### 3.2.3. Stage 2: Cross-Modal Fusion with Learned Gates

Once projected into a common space, the modalities are fused in two complementary ways: learned scalar gates that weigh each modality’s overall contribution, and cross-modal attention that captures fine-grained inter-modality relationships at each timestep.

**Learned gates.** Not all modalities are equally informative for operation classification. An accelerometer close to the spindle may carry strong cutting dynamics; a barometric pressure sensor may carry mostly session-level environmental drift. Rather than assuming uniform modality importance, each modality receives a learnable scalar gate that is trained jointly with the rest of the network:

(3) $$g_{i}=\sigma \left(w_{i}\right),\;{\tilde{S}}_{\mathrm{gated}}=\frac{\sum_{i=1}^{M}g_{i}\;\cdot \;{\tilde{H}}_{i}}{\sum_{i=1}^{M}g_{i}+\epsilon }$$

The gate values emerge from training: modalities that consistently improve classification receive higher weights; modalities that introduce noise or confounds receive lower weights. This is a soft, differentiable version of the manual sensor selection that practitioners currently perform by hand.

**Cross-modal attention.** The gates capture global modality importance but do not model how information should flow between modalities at specific timesteps. Cross-modal attention is designed to address this: the mean across all modality projections forms the query, and all modality-timestep pairs form the keys and values. The attention mechanism could thereby learn, for example, that at a direction-change timestep, the gyroscope and accelerometer signals should be weighted together, while at a quasi-steady pass, the acoustic signal carries more relative information; whether this capacity provides benefit at the current data scale is tested in Section 4.6:

(4) $$Q=\frac{1}{M}\sum_{i=1}^{M}{\tilde{H}}_{i},\;KV=\left[{\tilde{H}}_{1};\ldots ;{\tilde{H}}_{M}\right],\;A=\mathrm{MultiHead}\left(Q,KV,KV\right)$$

The fused output combines both mechanisms with layer normalization:

(5) $$H_{\mathrm{fused}}=\mathrm{LN}\left(\mathrm{LN}\left(Q+A\right)+{\tilde{S}}_{\mathrm{gated}}\right)$$

**Modality dropout.** During training, entire modality projections are stochastically zeroed with probability $p_{\mathrm{mod}}=0.1$, with at least one modality always retained. This regularization prevents the model from over-relying on any single modality, directly improving robustness when a sensor is unavailable or degraded at inference time—a realistic scenario in deployed CNC monitoring where sensors may lose connectivity or fail.

#### 3.2.4. Stage 3: Denoising Transformer Autoencoder

CNC sensors operate in a mechanically and electrically harsh environment. Spindle motors generate electromagnetic interference; cutting forces produce vibration that couples structurally between sensors; thermal gradients develop over the course of a machining session; and wireless transmission introduces occasional packet loss and dropouts. A representation learned without accounting for these noise sources fits the specific noise pattern of the training sessions and generalizes poorly to new conditions.

The denoising autoencoder provides a self-supervised learning signal that forces the fused representation to be noise-invariant. During training, the fused features are deliberately corrupted by adding Gaussian noise and randomly masking a fraction of positions:

(6) $${\tilde{H}}_{\mathrm{noisy}}=\left(H_{\mathrm{fused}}+N\left(0,\sigma^{2}\right)\right)⊙\left(1-m\right),\;m_{t,j}∼\mathrm{Bernoulli}\left(0.1\right)$$

The model must then reconstruct the original clean fused features from this corrupted input. A 2-layer Transformer encoder compresses the corrupted representation into a latent code z; a matching 2-layer Transformer decoder reconstructs the clean features by attending to z:

(7) $$z=\mathrm{TransformerEncoder}\left({\tilde{H}}_{\mathrm{noisy}}\right),\;{\hat{H}}_{\mathrm{recon}}=\mathrm{TransformerDecoder}\left(0,z\right)$$

Decoder queries are initialized to zero so that reconstruction at every position is driven purely by cross-attention to the encoder’s latent representation, without any autoregressive or positional prior. The reconstruction loss provides a secondary training objective:

(8) $$L_{\mathrm{recon}}=\mathrm{MSE}\left({\hat{H}}_{\mathrm{recon}},H_{\mathrm{fused}}\right)$$

The key consequence is that the latent code z must capture structure that persists across noise instances—the stable, operation-discriminative patterns in the sensor streams—rather than encoding noise-specific features that would not generalize.

#### 3.2.5. Stage 4: Temporal Modeling

The Transformer encoder in the denoising autoencoder applies global self-attention: every timestep attends equally to every other timestep within the window. This is effective for learning relationships between parts of the sequence but does not impose the causal, left-to-right structure of how a machining operation actually unfolds. A plunge entry at the start of a pocket operation is causally prior to the lateral clearing that follows; this ordering is meaningful for classification.

A 2-layer unidirectional Long Short-Term Memory network processes the latent sequence z to capture this causal temporal structure:

(9) $$H_{\mathrm{lstm}}=\mathrm{LN}\left(\mathrm{LSTM}\left(z\right)\right)\in R^{T\times d_{\mathrm{model}}}$$

The unidirectional (not bidirectional) configuration is a deliberate choice for two reasons. First, it preserves causal structure: each timestep’s hidden state is a function only of past observations, which is required for streaming inference where future sensor data is not yet available. Second, the combination of Transformer (global attention for cross-timestep relationships) and Long Short-Term Memory (causal sequential integration) exploits complementary strengths: the Transformer captures which timesteps are most relevant to each other; the Long Short-Term Memory integrates information sequentially as the operation progresses.

#### 3.2.6. Stage 5: Classification Head and Training Objective

Classification uses the hidden state at the last valid timestep, which summarizes the entire observed sequence:

(10) $$\hat{y}=\mathrm{softmax}\left(W_{\mathrm{cls}}H_{\mathrm{lstm}}\left[t_{\mathrm{last}},:\right]+b_{\mathrm{cls}}\right)\in R^{9}$$

The total training loss combines classification and reconstruction objectives:

(11) $$L=L_{\mathrm{cls}}+\lambda L_{\mathrm{recon}},\;\lambda =0.1$$

where $L_{\mathrm{cls}}$ is cross-entropy with label smoothing [34] (ϵ=0.1) and inverse-frequency class weights to address the class imbalance in damage classes (4–5 files each versus 15–20 for other classes). The reconstruction weight λ=0.1 is set low enough that the denoising objective regularizes the representation without dominating the classification signal.

The model also produces a 128-dimensional embedding via an attention-pooled projection head (Appendix C), used for the embedding visualizations in Section 4.

#### 3.2.7. Parameter Count

Table 3 summarizes the parameter budget. With 7 modality encoder groups at $d_{\mathrm{model}}=256$, the active components total approximately 5.1 million parameters.

### 3.3. Training Configuration

All models are trained with AdamW [35] at a learning rate of $10^{-3}$, batch size 32, and early stopping after 40 epochs of no validation improvement. The learning rate follows a linear warmup over 10 epochs, then cosine annealing with warm restarts [36]. Full hyperparameters are listed in Appendix A (Table A2).

All experiments ran on a workstation with dual NVIDIA RTX A6000 GPUs (48 GB each) using Python 3.10, PyTorch 2.5, CUDA 12.1, and Ubuntu Linux. Neural models trained on a single GPU. Traditional baselines ran on CPU using scikit-learn [37] and XGBoost [38].

### 3.4. Experimental Setup

Data were collected from a Bantam Tools Explorer desktop Computer Numerical Control mill, instrumented with 16 Arduino Nano 33 BLE Sense Lite boards. Each board captures inertial, environmental, and acoustic signals across 17 channels. Full hardware specifications are provided in Appendix A. Figure 2 shows sensor mounting locations across the machine structure.

#### 3.4.1. Sampling Configuration

Inertial channels sample at 119 Hz, environmental channels at 10 Hz, and electrical channels at 1 kHz. All streams are synchronized to the G-code execution timeline at an effective aligned rate of approximately 4 Hz. Full sampling details and a note on bandwidth limitations are provided in Appendix A.

#### 3.4.2. Sensor Consistency and Selection

Of the 16 deployed sensors, 6 maintain ≥93% data consistency across all files and are used for cross-validated experiments. These six span three structural groups: frame (3), spindle (1), and bed (2), providing spatial diversity across the machine’s primary vibration transmission paths. Full consistency data are provided in Appendix A (Table A1).

#### 3.4.3. Data Cleaning

Two cleaning steps reduce the 135 raw files to 120 analysis-ready files: sensor consistency filtering (files missing any of the six required sensors are excluded) and trailing NaN truncation (one file with 79% NaN-padded rows is truncated to its last valid reading). Full details are provided in Appendix A.

#### 3.4.4. Data Collection Protocol

Data were collected across two separate days to capture session-to-session variation. The resulting day-to-day environmental differences are analyzed as a potential confound in Section 4.3. Each toolpath strategy was executed under three conditions. Air-cutting ran the spindle with no material engagement. Active cutting engaged ultra-high-molecular-weight polyethylene at 0.635 mm axial depth. Damaged-spindle air-cutting removed one drive band to induce spindle runout, simulating drive-train degradation.

All operations used a 2-flute, 6.35 mm diameter high-speed steel endmill at 12,000 RPM and 1016 mm/min feed rate. Ultra-high-molecular-weight polyethylene was selected as the workpiece material for its consistent cutting behavior and minimal tool wear over repeated trials.

### 3.5. Modality Structure

Each of the six sensors captures 17 channels across 8 physical measurement types. Grouping these channels by physical modality, rather than by sensor unit, reflects the intuition that channels sharing the same measurement type share noise characteristics and dynamic range. This grouping defines the encoder’s modality structure. The three low-bandwidth environmental channels (pressure, temperature, proximity) share a single encoder group because they exhibit similar scalar dynamics at 10 Hz. The remaining five channel types (accelerometer, gyroscope, magnetometer, color, and root mean square audio) each receive their own encoder group. A seventh group handles the 8 machine-level electrical channels. This yields 110 input features across 7 encoder groups (Table 4).

### 3.6. Baseline Models

Five baselines are selected to isolate the contribution of individual architectural choices in MM-DTAE-LSTM. Each baseline removes a specific layer of complexity. Tree-based models test whether the multimodal structure and temporal modeling are necessary at all. The Multilayer Perceptron tests whether a flat feature vector is sufficient, without any sequential modeling. SimpleLSTM isolates the recurrent temporal component by removing cross-modal fusion, denoising, and attention.

- **XGBoost** [38]: Gradient-boosted decision trees on flattened window features.
- **Random Forest** [39]: Ensemble of 200 decision trees on flattened features.
- **Logistic Regression**: L2-regularized multinomial logistic regression on flattened features.
- **Multilayer Perceptron**: Three-layer feedforward network (512–256–128) with batch normalization, rectified linear unit activation, and dropout (0.3).
- **SimpleLSTM**: Two-layer unidirectional Long Short-Term Memory (256 hidden units) with dropout (0.3) and last-timestep classification. This baseline isolates the contribution of temporal modeling while removing cross-modal fusion, denoising, and attention.

Traditional baselines (XGBoost, Random Forest, Logistic Regression) operate on flattened window features (T×F reshaped to T·F), discarding temporal structure. Neural baselines (MLP, SimpleLSTM) receive the same windowed sequences as MM-DTAE-LSTM. All neural baselines share the same optimizer, label smoothing, gradient clipping, batch size, and early stopping schedule (Table A2), ensuring that differences in performance reflect architecture rather than training configuration.

### 3.7. Evaluation Protocol

#### 3.7.1. Five-Fold File-Level Stratified Cross-Validation

Sliding-window segmentation over sensor time series produces heavily overlapping segments. Adjacent windows share most of their timesteps. A random window-level split therefore leaks near-duplicate segments across train and test partitions, inflating accuracy estimates. To prevent this, all windows from a single operation file are assigned to exactly one partition.

Within each class, files are sorted alphabetically and assigned to folds by deterministic cyclic allocation:

(12) fold(f)=(sorted_index(f)mod5)+1

For fold *k*, the test set contains files in fold *k*, the validation set contains files in fold (kmod5)+1, and the training set contains all remaining files. We report mean ± standard deviation across the 5 folds.

#### 3.7.2. Data Preprocessing

Each fold’s preprocessing pipeline runs independently, with all statistics computed on training data only to prevent leakage.

1. **Feature extraction, filtering, and imputation.** Continuous sensor and electrical channels are extracted. Metadata columns that would cause leakage (machine position, feed rate, G-code text) are excluded. Three automated filters remove uninformative channels: channels with more than 50% missing values, zero-variance channels, and one member of each highly correlated pair (|r|>0.95), retaining the higher-variance member. For feature ablation experiments, modality groups are excluded at this stage per Table 5. Remaining missing values are imputed by forward-fill, back-fill, then zero-fill.
2. **Outlier clipping and normalization.** Values outside $Q_{1}-3\;\cdot \;\mathrm{IQR}$ and $Q_{3}+3\;\cdot \;\mathrm{IQR}$ are clipped before scaler fitting. A robust scaler (median centering, interquartile range scaling) is fitted on training-fold data only and applied identically to validation and test folds. Robust scaling is chosen because sensor distributions exhibit heavy tails from dropout recovery transients that standard normalization amplifies.
3. **Sliding window segmentation.** Normalized time series are segmented into overlapping windows of configurable size (16–256 timesteps) with configurable stride. Only complete windows are retained. Each window receives a majority-vote G-code operation label mapped to one of the nine classes.

### 3.8. Feature Ablation Design

The ablation removes sensor modalities in a fixed order determined by expected discriminative value. Proximity sensors produce near-constant output under the machine enclosure and carry no machining dynamics. Barometric pressure varies between collection sessions due to atmospheric drift, not machining activity. Color channels encode gantry position via LED shadow patterns but are sensitive to ambient lighting conditions. Magnetometer channels capture spindle motor fields but overlap substantially with accelerometer information for operation classification. Table 5 lists the five cumulative configurations produced by this ordering.

The minimal configuration retains 56 features: accelerometer, gyroscope, temperature, root mean square audio, and electrical channels. These are the modalities most directly tied to machining dynamics and the most practically deployable in a cost-constrained monitoring system.

### 3.9. Temporal Resolution Ablation

Window size controls the tradeoff between temporal context and inference latency. Shorter windows enable lower-latency streaming inference but capture fewer toolpath cycles. Longer windows cover more complete geometric trajectories but reduce training sample count per file and increase computational cost. We evaluate 10 window and stride combinations spanning 16 to 256 timesteps, corresponding to approximately 4 to 64 s at the 4 Hz aligned sampling rate. The full set of configurations is listed in Appendix A (Table A3).

## 4. Results

This section reports results in the order of the four research questions posed in the Introduction. Section 4.1 describes training convergence and the structure of learned representations. Section 4.2 and Section 4.3 report feature ablation results and the confound analysis that explains them. Section 4.4, Section 4.5 and Section 4.6 cover temporal resolution, model comparison, and architectural ablation. Section 4.7 and Section 4.8 report per-class performance and confusion structure.

### 4.1. Training Behavior and Learned Representations

**Figure A1:**
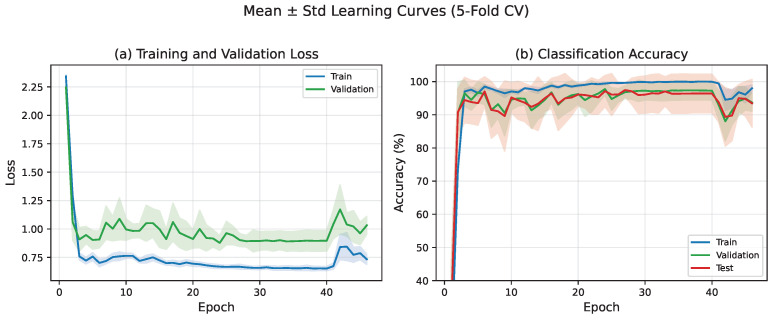
Averaged training and validation learning curves for MM-DTAE-LSTM at the best configuration (no proximity and pressure channels, window = 64, stride = 16), with ±1 standard deviation shading. (**a**) Loss converges within approximately 30 epochs with a modest generalization gap. (**b**) Training accuracy saturates near 100% while validation and test accuracy plateau near 95%, indicating mild overfitting consistent with a 120-file dataset.

**Figure A2:**
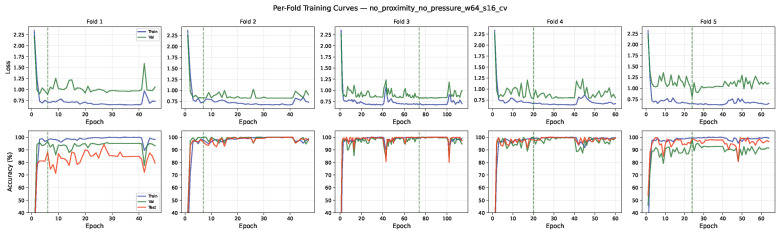
Per-fold training curves for MM-DTAE-LSTM at the best configuration (no proximity and pressure channels, window = 64, stride = 16). Top row: loss; bottom row: accuracy (train, validation, test). Folds 1, 2, 4, and 5 converge within 40–60 epochs; Fold 3 trains to approximately 100 epochs before early stopping, reflecting greater difficulty in that fold’s particular train/test partition. Fold-to-fold variance in final test accuracy (85–100%) is driven primarily by the uneven distribution of low-count damage classes across folds.

Training converges quickly at the best configuration (no proximity and pressure, window = 64, stride = 16). Within approximately 30 epochs, training accuracy saturates near 100% and validation and test accuracy plateau near 95%. The modest generalization gap is consistent with a 120-file dataset. Averaged learning curves appear in Figure A1. Per-fold curves appear in Figure A2 (Appendix B).

**Figure 4:**
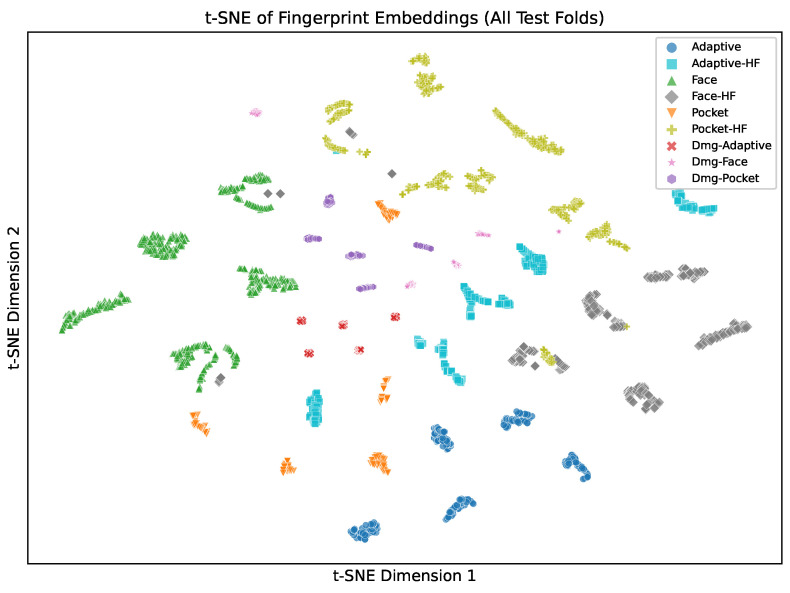
t-distributed Stochastic Neighbor Embedding visualization of 128-dimensional fingerprint embeddings from MM-DTAE-LSTM, pooled across all 5 test folds (3479 samples; perplexity = 30). Colors indicate class; marker shapes indicate toolpath strategy. Air-cut classes form tight clusters; active-cut classes cluster near their air-cut counterparts; damage classes partially overlap their air-cut counterparts.

**Figure A6:**
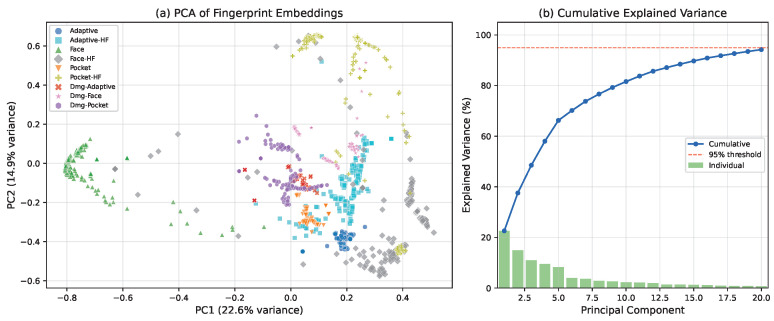
Principal component analysis [41] of 128-dimensional fingerprint embeddings (3479 test samples across 5 folds). (**a**) First versus second principal component scatter shows linear separability among the three toolpath families. (**b**) Cumulative explained variance: 21 components capture 95% of embedding variance; components 1 through 5 account for 66.2%.

The learned representations reflect the physical structure of the task. Figure 4 shows 128-dimensional fingerprint embeddings pooled from test sets across all 5 folds (3479 samples). Air-cut classes form tight, well-separated clusters. Active-cut classes cluster near their corresponding air-cut toolpaths, reflecting shared trajectory geometry with additional cutting-force signatures. Damage classes partially overlap their air-cut counterparts, consistent with spindle degradation producing subtle shifts within the same toolpath family. Toolpath geometry is the dominant organizing principle in embedding space. Operating condition provides a secondary separation within each family. Principal component analysis of the same embeddings (Figure A6, Appendix B) confirms this structure is not a nonlinear artifact. The first two principal components capture 37.6% of total embedding variance, with toolpath families forming visually distinct regions.

**Figure 5:**
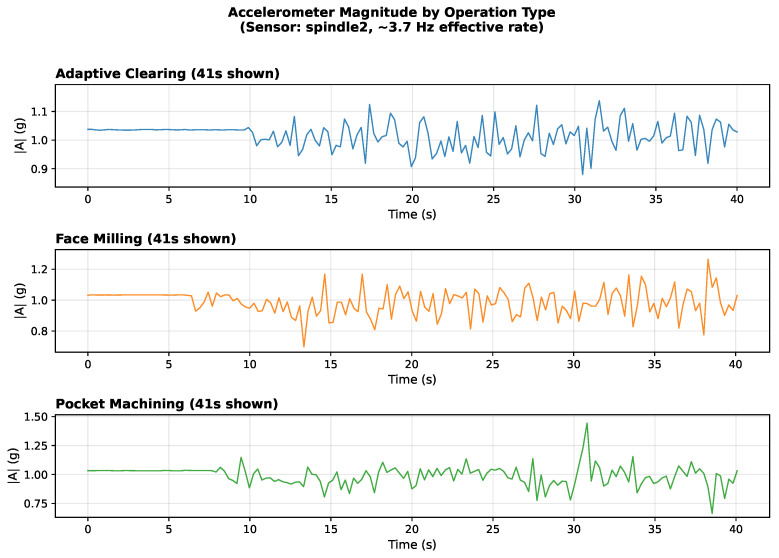
Accelerometer magnitude signatures for three air-cut toolpath strategies (spindle sensor). Adaptive clearing shows periodic direction-change patterns; face milling exhibits steady-state gantry motion; pocket machining displays intermittent plunge and lateral transitions.

The discriminative structure in the embeddings has a physical basis visible in the raw sensor signals. Figure 5 shows accelerometer magnitude signatures for the three air-cut toolpath strategies. Adaptive clearing produces periodic direction-change transients. Face milling exhibits quasi-steady gantry motion. Pocket machining alternates between axial plunge and lateral clearing signatures. These differences are present during air-cutting, confirming that toolpath geometry alone produces discriminative sensor signatures without material engagement.

### 4.2. Feature Ablation Results

The encoder maintains strong performance as sensors are removed, while simpler models degrade substantially. Table 6 presents classification accuracy across all five feature configurations at the default temporal resolution.

Three patterns stand out. First, removing proximity and pressure channels improves the encoder by 2.8 points, from 93.5% to 96.3%. These channels carry session-level environmental variation rather than machining dynamics, as the confound analysis in Section 4.3 demonstrates. Second, from this 98-feature peak to the minimal 56-feature configuration, the encoder loses only 3.8 points. XGBoost loses 10.7 points across the same range and Logistic Regression loses 7.2 points. Third, neural baselines (MLP and SimpleLSTM) show intermediate robustness, losing 3.0 and 3.8 points, respectively. The denoising objective provides robustness beyond what temporal modeling alone delivers.

### 4.3. Confound Analysis

**Figure 6:**
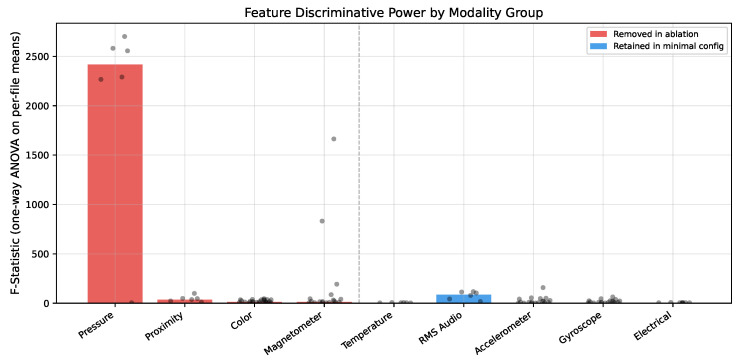
Distribution of analysis of variance *F*-statistics by modality group. Red bars indicate modalities removed during feature ablation; blue bars indicate retained modalities. Individual feature *F*-values overlaid as scatter points. Pressure channels (off-scale, *F* > 2000) dominate due to session confounds rather than machining physics.

To understand why removing pressure and proximity channels improves the encoder, we performed a one-way analysis of variance (ANOVA) on per-file summary statistics across the nine operation classes (${\mathrm{df}}_{\mathrm{between}}=8$, ${\mathrm{df}}_{\mathrm{within}}=111$; group sizes range from n=4 to n=20). One-way ANOVA was selected because the analysis tests whether the group means of a single continuous feature differ across operation classes, which is a standard use case for this method. All tests were implemented using scipy.stats.f_oneway (SciPy v1.11 [40]). Significance is assessed at α=0.05 with Bonferroni correction for 110 simultaneous tests ($\alpha_{\mathrm{adj}}=4.5\times 10^{-4}$); all features reported as significant remain so after correction. Table 7 summarizes results by modality group and Figure 6 shows the distribution of *F*-statistics by modality.

Pressure channels produce the highest *F*-statistics in the entire dataset (*F* > 2000, $\eta^{2}>0.99$), far exceeding accelerometer and gyroscope channels that encode actual machining dynamics. This is not because pressure is informative for CNC monitoring. Barometric pressure varied between collection days (∼100.0 kPa on day one versus ∼101.5 kPa on day two), and certain operation classes were disproportionately collected on specific days. The pressure sensor encodes *when* data were collected, not *what* was being machined. Within-class standard deviation is only ∼0.05 kPa, confirming that the apparent discriminative power is entirely a session-level artifact. Proximity channels produce similarly inflated *F* values from near-constant readings that differ by sensor placement rather than machining activity.

This confound directly explains the asymmetric degradation seen in Table 6. Tree-based models exploit high-*F* features that provide easy class separation through environmental shortcuts. Removing proximity and pressure drops XGBoost by 2.7 points. Removing color and magnetometer costs another 8.0 points, for a total drop of 10.7 points (95.1% to 84.4%). The encoder learns representations primarily from lower-*F* channels (accelerometer and gyroscope: median F≈11, $\eta^{2}\approx 0.44$) and maintains 92.5% at the minimal 56-feature configuration. A model that appears accurate at full features may be exploiting session-level variation that does not generalize to new collection days or deployment environments.

This confound is a direct consequence of the two-day collection protocol, in which operation classes were not balanced across collection days. Future studies should randomize operation-day assignments or collect all classes on every collection day. This design lesson applies to any multi-session sensor dataset, not only to CNC monitoring.

### 4.4. Temporal Resolution Results

Classification accuracy is stable across a wide range of window sizes (Figure A10, Appendix B).

**Figure A8:**
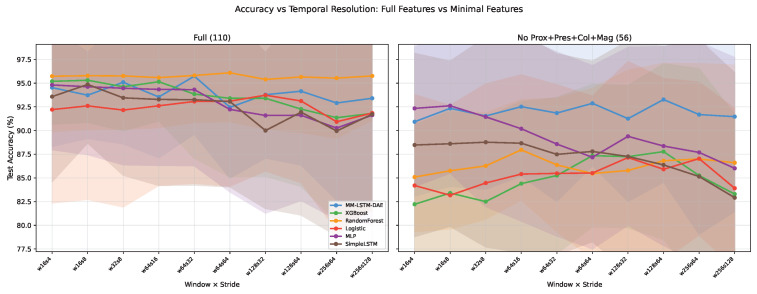
Classification accuracy comparison between full (110 features) and minimal (56 features) configurations across all ten temporal resolutions. The encoder maintains consistent performance under sensor reduction while tree-based baselines exhibit substantial degradation at the minimal feature set.

**Figure A9:**
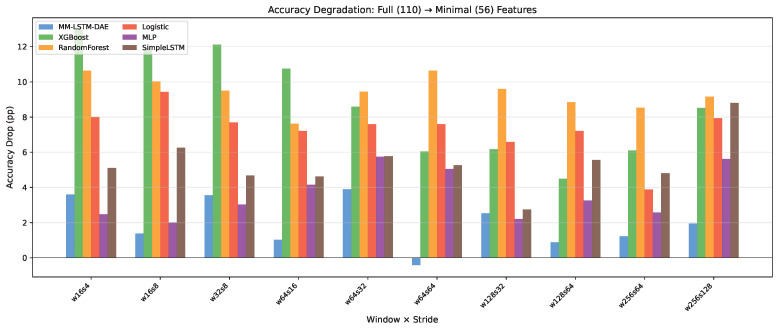
Accuracy degradation (full minus minimal, in percentage points) per model across temporal resolutions. XGBoost suffers the largest degradation (8–11 points across window sizes), while the encoder consistently degrades least (under 3 points at most configurations).

**Figure A10:**
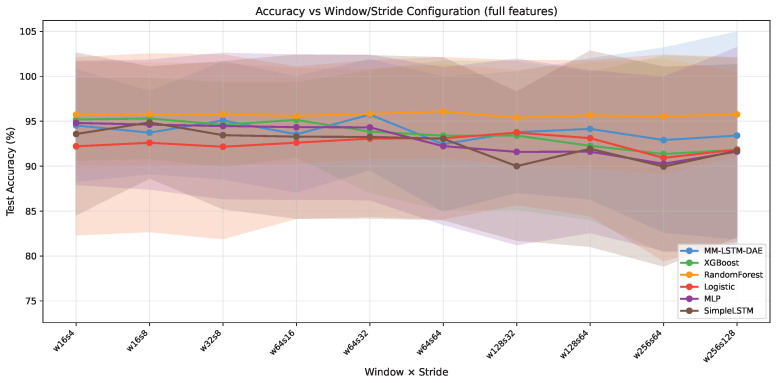
Classification accuracy as a function of window size for each model (full 110-feature configuration). Error bars show ±1 standard deviation across 5 folds. The encoder and neural baselines peak at medium windows (32–64), while Random Forest remains remarkably stable across all window sizes.

The encoder varies by only 3.2 points across all ten temporal configurations (92.5% to 95.7%), a spread modest relative to fold-to-fold variance. At the 4 Hz aligned sampling rate, a 64-timestep window spans approximately 16 s and covers several complete direction-change or step-over cycles for all three toolpath strategies. Medium windows (32–64 timesteps) provide the best balance of temporal context and training sample count. Large windows (w=256) increase fold-to-fold variance because fewer windows are available per file, amplifying the class imbalance in damage categories. Random Forest maintains 95.5–96.1% regardless of window size, confirming that its feature-level splits do not depend on temporal extent. Full versus minimal feature comparison and per-model degradation plots appear in Figure A8 and Figure A9 (Appendix B).

### 4.5. Model Comparison

At the best configuration, MM-DTAE-LSTM leads all five baselines in both accuracy and macro F1 and achieves the lowest fold-to-fold variance. Table 8 reports results at the encoder’s best feature configuration (no proximity and pressure, 98 features).

The accuracy differences (3.1–5.2 percentage points) correspond to small-to-medium Cohen’s *d* effect sizes (0.45–0.74). All 95% confidence intervals span zero given *n* = 5 folds, which is expected at this sample size. Two sources of evidence support the encoder’s advantage beyond this single-configuration snapshot. First, the direction of advantage is consistent across all five baselines at this configuration. The probability that this consistent direction arises by chance is ${\left(1/2\right)}^{5}=3.1\%$. Second, the advantage grows under sensor reduction. At 56 features, the encoder leads all five baselines by 2.3–8.1 points, a regime where tree-based and linear models degrade sharply.

The encoder achieves the lowest fold-to-fold variance (standard deviation 4.7% accuracy, 8.0% macro F1), compared to 6.3–11.5% and 10.4–14.6% for baselines. High fold-to-fold variance means reliability in deployment depends on which operation files are withheld. Low variance is therefore a practical advantage beyond mean accuracy.

Training takes approximately 3.3 min per fold on a single GPU, comparable to XGBoost on CPU (approximately 3.2 min) and substantially faster than SimpleLSTM (approximately 13 min). The faster convergence relative to SimpleLSTM is attributed to the denoising reconstruction loss, which stabilizes optimization and enables earlier stopping. Timing details appear in Table A4 (Appendix B).

### 4.6. Architectural Component Ablation

To isolate which components of MM-DTAE-LSTM drive performance, we evaluated seven architectural configurations at the best feature and temporal settings (98 features, window = 64, stride = 16). Each configuration removes or simplifies one component. Table 9 reports mean accuracy and macro F1 across five folds. Following inferences are made based on the observation of each component’s performance within the architecture:

- **The denoising objective is the single most important component.** Removing noise injection, masking, and reconstruction loss while keeping the Transformer architecture costs 4.4 points in accuracy and 7.9 points in macro F1, the largest degradation of any ablation. The impact concentrates on damage classes: dmgFac F1 drops from 70.8% to 40.3% and dmgPoc from 78.6% to 57.6%. Denoising provides a regularization benefit most valuable for low-data, harder-to-discriminate conditions. Removing the entire Transformer block costs only 1.7 points in F1, far less than removing the denoising objective while keeping the Transformer. The denoising training signal is what matters, not the Transformer architecture that hosts it.
- **Cross-modal attention does not improve performance on this dataset.** Removing cross-modal attention while keeping all other components yields 97.4% accuracy and 93.2% macro F1, both higher than the full model. The dataset (120 files, 9 classes) is likely too small for cross-modal attention to learn reliable inter-modality relationships. The gated modality-weighted average provides sufficient fusion at this data scale. Whether cross-modal attention benefits larger datasets remains to be tested.
- **The minimal LSTM-only model is competitive.** A single shared projection followed by concatenation and LSTM classification achieves 93.9% accuracy and 89.7% macro F1, only 2.1 and 2.4 points below the full model. Much of the classification signal is resolved by temporal modeling alone. The denoising objective accounts for the incremental gain that separates the full model from simpler alternatives.
- **Model capacity does not drive performance.** To assess whether the encoder’s parameter budget is justified, we evaluated five model dimensions spanning a 220-fold range in active parameter count at the same best configuration (Table 10).

Classification accuracy is stable across this 220-fold parameter range (Table 10). One-way ANOVA confirms no significant effect of model dimension on either accuracy (F(4,20)=0.046, p=0.996) or macro F1 (F(4,20)=0.151, p=0.961). No pairwise comparison reaches significance at α=0.05. The smallest configuration ($d_{\mathrm{model}}=32$, 0.1M parameters) achieves 97.6% accuracy; the best ($d_{\mathrm{model}}=64$, 0.4M parameters) achieves 98.5% accuracy and 96.4% macro F1, both exceeding the default $d_{\mathrm{model}}=256$. Increasing to 512 (21.9 M parameters) provides no improvement. Combined with the component ablation above, these results confirm that the denoising training objective, not model size or architectural complexity, is the primary driver of the encoder’s advantage over baselines.

### 4.7. Per-Class Performance

Performance varies substantially across the nine classes. Well-represented classes are classified reliably. Damage classes cannot be evaluated reliably at current data volumes. Full per-class accuracy and F1 scores appear in Table A5 and Table A6 (Appendix B).

For classes with 15 or more training files, the encoder achieves at least 93.6% accuracy and at least 94.0% F1. The three air-cut classes (adaptive, face, pocket) are the most reliably classified, reflecting the clean and distinct motion signatures visible in Figure 5. Active-cut classes share toolpath geometry with their air-cut counterparts but add cutting-force signatures, making within-toolpath condition discrimination harder.

Damage class results should be treated as preliminary observations rather than reliable estimates. With only 4–5 files per class across 5 folds, some folds contain zero test samples from these classes. The reported values (damageface F1 = 65.8 ± 39.4%, damagepocket F1 = 73.6 ± 43.4%) reflect this extreme variance rather than true model capability. All models struggle comparably with damage classes. Data quantity, not model capacity, is the bottleneck. Meaningful damage-class evaluation requires at least 10 files per class.

### 4.8. Confusion Matrices

**Figure 7:**
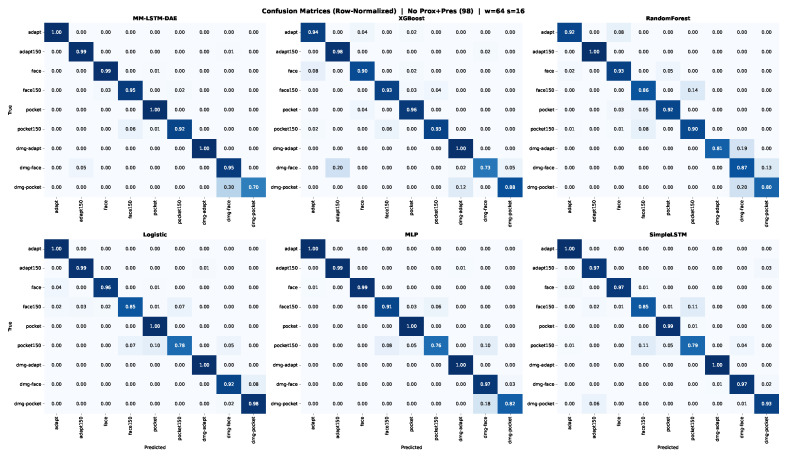
Row-normalized confusion matrices aggregated across 5 folds for all six models at the best configuration (no proximity and pressure, window = 64, stride = 16). The encoder achieves near-diagonal structure for air-cut and active-cut classes; damage classes show diffuse misclassification across all models.

The confusion matrices reveal the structure of the errors, not just the aggregate accuracy. Figure 7 shows row-normalized confusion matrices aggregated across 5 folds for all six models.

Misclassifications occur almost exclusively within toolpath families, never across them. damagepocket is confused with pocket and pocket150025 but never with face or adaptive classes. This pattern holds across all six models. Toolpath geometry is therefore the dominant organizing principle in the sensor data. Even during air-cutting, adaptive clearing, face milling, and pocket machining produce sufficiently distinct motion signatures that cross-family errors are rare. Within-family condition discrimination, separating air-cut, active-cut, and damaged-spindle variants of the same toolpath, is the harder subtask. This is consistent with the wider spread of damage-class embeddings visible in Figure 4.

Taken together, the results answer the four research questions as follows. Accelerometer, gyroscope, acoustic, and electrical channels carry genuine discriminative signal; pressure channels encode a session-level atmospheric confound (F>2000) rather than machining physics and degrade performance when retained. The encoder degrades substantially less than tree-based baselines under sensor reduction (3.8 points versus up to 10.7 points), and this robustness is concentrated in the denoising objective rather than the architectural complexity. The denoising objective is the critical component: removing it costs 7.9 macro-F1 points, compared to 1.7 points for removing the entire Transformer block. The minimum viable sensor configuration is the 56-feature set using only inertial measurement unit boards, a microphone, and electrical channels, achieving 92.5% accuracy at approximately USD 492 in hardware.

## 5. Discussion

This section interprets the results in the context of the four research questions. Section 5.1 explains what the sensor ablation reveals about modality contributions. Section 5.2 defines when a lightweight model is preferable to the encoder. Section 5.3 discusses implications for process monitoring deployment. Section 5.4 provides concrete deployment recommendations. Section 5.6 states the study’s limitations.

### 5.1. What the Sensor Ablation Actually Tells Us

Three modality groups carry the discriminative signal. Accelerometers and gyroscopes encode structural vibration and rotational dynamics produced by different toolpath motions. Root mean square audio captures acoustic emission from material engagement and separates air-cut from active-cut conditions. Electrical channels reflect spindle current and voltage, which respond directly to cutting load. These three groups together define the 56-feature minimal configuration that achieves 92.5%.

Each removed modality fails for a distinct physical reason. Proximity sensors produce near-constant readings that encode sensor placement rather than machining activity. Pressure encodes session-level atmospheric variation, as confirmed by ANOVA *F*-statistics above 2000 at every sensor location. Color channels capture reflected light intensity that varies with gantry position as the machine structure moves relative to a fixed LED source. This positional information is already implicit in the acceleration sequence, making the color contribution marginal. Removing these channels costs only 1.6 percentage points in the ablation. Magnetometer channels are partially redundant with accelerometer channels for this classification task, though they may carry independent signal for other monitoring objectives.

The pressure confound identified here is the most severe case, but the session-level day effect may extend to other channels in a weaker form. Magnetometer channels on frame_r2 show elevated *F*-statistics (F>800) that likely reflect a spatially consistent magnetic field gradient at that location rather than machining dynamics. Temperature channels show modest *F*-values (F≈4.5) that likely reflect session-level thermal variation rather than per-operation physics. Practitioners should treat any channel with anomalously high *F*-statistics relative to its modality group as a candidate confound rather than a discriminative feature. Future validation studies should use session-stratified cross-validation, where entire recording sessions are held out, rather than file-level splits, to detect this class of confound before deployment.

The minimal 56-feature configuration has a direct hardware implication. A CNC machine can be retrofitted with only inertial measurement unit boards, a microphone, and a current-voltage data acquisition module. Total hardware cost is approximately USD 492 (Table 11), and no controller modification is required.

### 5.2. When to Use a Lightweight Model vs. the Encoder

At full sensor coverage (110 features), Random Forest achieves 95.6% and XGBoost 95.1%, both comparable to or slightly above the encoder’s 93.5%. This is not a failure of the encoder. The full-feature dataset includes confound-prone channels that tree-based models exploit effectively. Under these conditions, the simpler model is a reasonable choice. It trains faster, is easier to deploy, and does not require gradient optimization.

The encoder’s advantage emerges in two specific regimes. Under sensor reduction, at 56 features the encoder (92.5%) leads every baseline including the Multilayer Perceptron (90.2%). Tree-based models degrade sharply. Random Forest drops to 88.0% and XGBoost to 84.4%. In terms of fold-to-fold consistency, the encoder achieves the lowest variance across folds (standard deviation 4.7%), compared to 6.3–11.5% for baselines. A model that performs well on average but varies widely across operation files is unreliable in deployment.

The architectural ablation (Section 4.6) clarifies whether 5.1 million parameters are justified for 120 files and nine classes. The parameter budget is not uniformly justified. The denoising objective accounts for the bulk of the full model’s advantage over simpler alternatives. Removing the entire Transformer block costs only 1.7 points in macro F1. Removing the denoising objective while keeping the Transformer costs 7.9 points. A simpler architecture combining per-modality projection, modality-gated fusion, the denoising objective, and an LSTM would likely recover most of the full model’s performance at a fraction of the parameter count.

The cross-modal attention result further constrains architectural necessity. Removing cross-modal attention improves both accuracy and macro F1 on this dataset, indicating it adds complexity without benefit at this data scale. A model capacity ablation (Table 10) confirms this directly: reducing $d_{\mathrm{model}}$ from 256 to 64 shrinks the model from 5.1M to 0.4M active parameters while improving macro F1 from 94.0% to 96.4%. The useful capacity lies in the denoising training signal, not the parameter count.

Cut duration limits the temporal modeling advantage in this study. All operations run for approximately 1–3 min. At the approximately 4 Hz aligned sampling rate, each file yields roughly 240–720 timesteps. With a 64-timestep window, each file contributes only 15–30 non-overlapping windows. Under these conditions, classification reduces largely to recognizing the characteristic pattern of a short segment. The trochoidal jerk of adaptive clearing, the steady sweep of face milling, and the plunge signature of pocket machining are all contained within a single short window. The LSTM and Transformer components are designed to capture long-range dependencies that develop across hundreds of sequential timesteps. With 1–3 min cuts, there is not enough temporal extent per file for this capacity to be fully exercised. This explains why the minimal projection-then-LSTM model performs within 2.1 percentage points of the full architecture.

As cut duration grows toward the 10–30 min programs typical of production machining on complex parts, the temporal modeling advantage is expected to scale meaningfully. Longer programs produce more direction-change cycles, more step-over transitions, more plunge-clear alternations, and more variation in cutting conditions as the tool traverses different regions of the workpiece. These are precisely the sequential dependencies that the LSTM is designed to integrate and that the Transformer is designed to attend over. The current results represent a conservative lower bound on the temporal modeling benefit rather than a ceiling.

### 5.3. Implications for Process Monitoring

Distributed sensors can reliably identify what a CNC machine is doing from physical signals alone, without querying the controller. At 96.3% accuracy for well-represented classes and 92.5% with half the sensor channels removed, the approach is practical for benchtop process monitoring. Three properties of the learned representations are particularly relevant to deployment.

First, learned embeddings are operation-specific and tightly clustered. Mean intra-class cosine similarity (computed on the 20-component PCA projection capturing 95% of embedding variance) is 0.81±0.18. Mean inter-class centroid similarity is −0.08±0.18. Class centroids are nearly orthogonal in embedding space. The model produces consistent, distinctive signatures for each operation type, not just a classification decision.

Second, misclassifications are structurally interpretable. Errors occur within toolpath families, for example a pocket operation confused with another pocket condition, and never across families. Classification failures are therefore the most conservative possible errors. They preserve the toolpath identity while misidentifying the condition.

**Figure 8:**
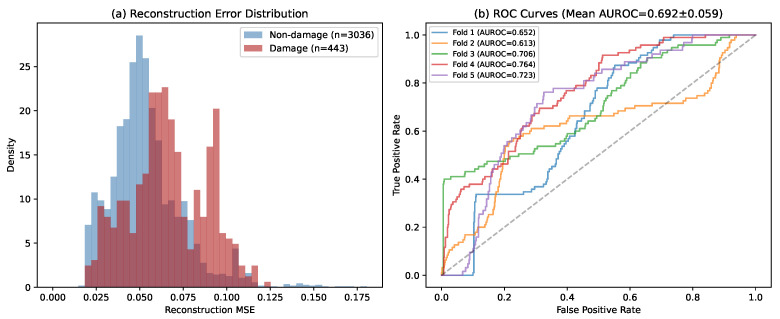
Reconstruction-error anomaly detection. (**a**) Distribution of per-window reconstruction mean squared error for non-damage (blue) and damage (red) test samples pooled across 5 folds. Distributions overlap substantially. (**b**) Per-fold receiver operating characteristic curves; mean area under the curve = 0.69±0.07.

Third, reconstruction-based anomaly detection proved ineffective (area under the receiver operating characteristic curve = 0.69±0.07; Figure 8). This is a negative but informative result. The denoising autoencoder’s reconstruction objective is optimized to reconstruct the fused modality embedding, which encodes toolpath geometry rather than spindle health. Reconstruction error reflects how well the model recognizes the operation type, not whether the spindle is degraded. Anomaly-sensitive representations require a dedicated health-aware training objective or explicit fault-condition supervision.

### 5.4. Deployment Recommendations

- **Feature configuration**: The no-proximity/no-pressure configuration (98 features) achieves the best accuracy and removes channels with no genuine discriminative value. For cost-constrained deployment, the minimal 56-feature configuration retains 92.5% accuracy using only inertial, acoustic, and electrical modalities.
- **Temporal resolution**: Window size 64 with stride 16–32 provides the best accuracy–latency tradeoff (approximately 16 s at 4 Hz). Stride 32 halves computational load with minimal accuracy reduction.
- **Model selection**: When all sensors are available and environmental conditions are stable, Random Forest provides approximately 96% accuracy with sub-second training. However, its performance relies partly on session-level environmental confounds; the encoder with the 56-feature minimal configuration is more robust to new collection sessions or changing environments.
- **Fault detection**: Damage-class monitoring requires at least 10 files per damage class for reliable performance. The current 4–5 files per damage class are insufficient for production-grade fault detection; this is a dataset limitation, not a model limitation.

### 5.5. Physical Interpretation of Sensor Modality Contributions

#### 5.5.1. Why Accelerometers Are Essential

Accelerometers capture broadband structural vibration along three orthogonal axes, encoding both the forced response from servo-driven axis motion and, during active cutting, the dynamic cutting force components transmitted through the machine structure. Even during air-cutting, toolpath strategies produce distinct acceleration profiles. Adaptive clearing generates high-jerk transients at trochoidal corners. Face milling produces quasi-periodic motion with acceleration pulses at step-over transitions. Pocket machining alternates between axial plunge and lateral clearing with bimodal frequency content. During active cutting, additional signatures emerge from cutting forces near the tooth-passing frequency. The tooth-passing frequency is above the 59.5 Hz inertial measurement unit Nyquist limit, but discriminative information persists in the low-frequency force envelope (structural resonance excitation at 10–50 Hz) and in modulation of servo tracking error by cutting load.

#### 5.5.2. Why Gyroscopes Complement Accelerometers

Gyroscopes measure angular rate about three axes, sensing phenomena not visible to accelerometers: spindle angular velocity fluctuations from belt slip or runout, gantry pitch and yaw during rapid direction reversals, and bed-mounted rotational modes excited by off-center cutting loads. This orthogonal physical basis, translational versus rotational dynamics, means each modality captures information the other cannot. Both are retained in the minimal configuration, and their similar median analysis of variance *F*-statistics (accelerometer: 10.8, gyroscope: 11.4) indicate comparable but non-redundant discriminative contributions. Isolating the individual contribution of each modality would require a finer-grained ablation not performed in this study, and is noted as a direction for future work.

#### 5.5.3. The Role of Acoustic Features

Root mean square audio captures airborne acoustic emission, which is physically distinct from structure-borne vibration measured by accelerometers. Air-cutting produces tonal spindle noise dominated by the belt-driven fundamental and its harmonics; active cutting adds broadband stochastic emission from chip formation, shearing, and tool-workpiece friction. This makes root mean square audio particularly discriminative for separating air-cut from active-cut conditions (median F=91, the highest among retained modalities), explaining why it is retained even in the minimal configuration.

#### 5.5.4. Temperature as a Slow-Response Channel

Temperature channels respond on timescales of minutes rather than milliseconds, encoding thermal equilibrium state rather than dynamic machining events. Their modest discriminative power (F≈4.5, comparable to electrical channels) likely reflects session-level thermal variation, including spindle warm-up duration and ambient workshop temperature, rather than per-operation physics. Their retention in the minimal configuration reflects the modality-group granularity of the ablation design rather than a demonstrated per-operation diagnostic value. Per-operation temperature analysis would require higher-frequency thermal sensing than the 10 Hz sampling available on these boards.

### 5.6. Limitations

1. **Single machine**: All data were collected from one Bantam Tools Explorer desktop Computer Numerical Control mill. Industrial machines differ substantially in structural stiffness, spindle drive type, and vibration propagation paths. The methodology transfers directly; accuracy values require per-machine revalidation.
2. **Single material and depth**: Active cutting used ultra-high-molecular-weight polyethylene at 0.635 mm depth only. Metals produce fundamentally different cutting forces, thermal loads, and acoustic emissions.
3. **Limited operation types**: Three computer-aided manufacturing strategies were tested. Drilling, threading, and multi-axis contouring were not tested.
4. **Controlled environment**: The laboratory setting has minimal external vibration and consistent ambient temperature. Production floors introduce floor-borne vibration, coolant dynamics, and tool wear progression as additional confounding factors.
5. **Class imbalance**: Damage classes have only 4–5 files versus 15–20 for air-cut and active-cut classes.
6. **Sampling bandwidth**: The approximately 4 Hz aligned rate captures only low-frequency dynamics. Higher-bandwidth acquisition (1–10 kHz) would provide access to tooth-passing harmonics and chatter frequencies.
7. **Model capacity**: Performance is stable across $d_{\mathrm{model}}\in \left\{32,64,128,256,512\right\}$, spanning a 220-fold parameter range (Table 10), with the smallest tested dimensions achieving the highest macro F1. The default $d_{\mathrm{model}}=256$ is overparameterized for this dataset; however, the minimum viable dimension under larger data scales remains untested.

## 6. Conclusions

CNC operation classification from distributed multimodal sensors is feasible on a benchtop platform. The sensor selection question has a clear answer from this study. Accelerometer, gyroscope, root mean square audio, and electrical channels carry the discriminative signal. Proximity and pressure channels, despite producing the highest ANOVA *F*-statistics in the dataset, encode session-level environmental variation rather than machining physics and should be excluded.

We introduced MM-DTAE-LSTM, a Multimodal Denoising Temporal Attention Encoder with LSTM-based classification, and evaluated it across 690 cross-validated experiments spanning five sensor-ablation levels, ten temporal resolutions, and five baseline models. Five findings stand out:

1. **The encoder is robust under sensor reduction.** From its 98-feature optimum (96.3%) to the minimal 56-feature configuration (92.5%), the encoder loses 3.8 points while XGBoost loses 10.7 points across the same range. This robustness is driven primarily by the denoising objective and represents the architecture’s primary practical advantage over simpler models.
2. **High *F*-statistics can be misleading.** Pressure channels produce the highest ANOVA *F*-statistics in the dataset (F>2000) while carrying no machining information. This confound, arising from a non-randomized two-day collection protocol, inflated baseline accuracy and would have gone undetected without the ablation study. Sensor selection based on univariate statistical tests alone is insufficient.
3. **Toolpath geometry is the dominant classification signal.** All models, including simple baselines, classify operation type from toolpath-induced motion patterns. Misclassifications occur within toolpath families (condition errors), not across them (toolpath errors). This holds during air-cutting, confirming that material engagement is not required for operation identification.
4. **Damage-class evaluation is not yet reliable.** With 4–5 files per damage class, performance estimates are dominated by cross-validation sampling variance rather than model capability. Expanding to at least 10 files per damage class is the most important data collection priority for future work.
5. **The denoising objective is the critical architectural component.** An architectural ablation across seven configurations shows that removing the denoising training signal costs 7.9 points in macro F1 and collapses damage-class performance (dmgFac F1 drops from 70.8% to 40.3%), while removing the Transformer block costs only 1.7 points. Removing cross-modal attention unexpectedly improves performance on this dataset. The minimal LSTM-only model sits within 2.4 points of the full architecture. The value of the full model lies in the denoising regularization, not the architectural complexity.

**Practical recommendations.** For maximum classification accuracy, the encoder with the 98-feature configuration should be deployed (no proximity or pressure) at window size 64 and stride 16. For cost-constrained or edge deployment, the minimal 56-feature configuration achieves 92.5% using only inertial measurement unit boards, a microphone, and an electrical data acquisition module. Total hardware cost is approximately $492, and no controller modification is required. When all sensors are available and the environment is stable, Random Forest achieves comparable accuracy with simpler training. When sensor coverage is limited or environmental conditions change, the encoder is the more robust choice.

Future work will address:

- **Longer-duration cuts to exercise temporal modeling capacity.** All operations in this study ran for 1–3 min, producing short per-file sequences at approximately 4 Hz that limit the temporal modeling advantage of the LSTM and Transformer components. Production machining programs on complex parts run for 10–30 min, generating far richer sequential structure across direction changes, step-overs, and material transitions. These are precisely the long-range dependencies these components are designed to capture. Evaluating on longer-duration cuts is expected to widen the performance gap between the full temporal architecture and simpler baselines.
- **Generalization across machines and materials.** Transfer learning and few-shot fine-tuning to industrial machines across diverse materials (aluminum, steel, titanium), depths of cut, and extended operation taxonomies (drilling, threading, multi-axis contouring).
- **Expanded fault datasets.** Collecting at least 10 files per damage class with varied degradation modes (bearing wear, misalignment, tool breakage) to enable reliable production-grade fault detection.
- **High-bandwidth acquisition and edge deployment.** Direct inertial measurement unit sampling at 1–10 kHz to capture tooth-passing harmonics, paired with edge-optimized inference for continuous streaming process monitoring.
- **G-code sequence reconstruction.** Extending the architecture from operation classification toward full G-code sequence prediction, recovering commanded toolpath, feed rate, and spindle parameters directly from sensor signals for complete program verification.

## Figures and Tables

**Table 1:** Comparison with Recent Experimental Studies in Sensor-Based CNC/Machining Monitoring. CV = cross-validation; Accel. = accelerometer; Vib. = vibration; AE = acoustic emission; Op. = operation; Feat. = features.

Study	Task	Sensors	Classes	Best Metric	Method	Validation	Ablation
Janssens [11]	Bearing fault	Accel. (1)	4	93.6% acc	1D-CNN	Train/test	No
Bhandari [13]	Roughness	Sound + force (2)	4	92.7% acc	Transformer	50/25/25	No
Cao [16]	Weld penetr.	Acoustic + photo (2)	3	99.7% acc	Cross-attn	Repeated	No
Wang [17]	Tool wear	Force + vib + AE (7)	3	—	DTAE-ResNet	Train/test	No
Stathatos [12]	Turning qual.	Vib. + torque + speed (8)	4	$F_{1}\;>\;0.97$	1D-CNN	10-fold CV	No
Coelho [28]	CNC anomaly	Current + vib (3)	2	91.6% acc	1D-CNN	80/10/10	No
Kimmell [29]	Attack detect.	Energy (1)	2	—	Threshold	Experimental	No
Ours	Op. classif.	6 sensors (110 feat.)	9	96.3% acc	MM-DTAE-LSTM	5-fold CV	Yes

**Table 2:** Nine-Class Classification Task.

Toolpath	Condition	Class Label	Files
Adaptive Clearing	Air-cut	adaptive	20
Adaptive Clearing	Active-cut	adaptive 150025	17
Adaptive Clearing	Damage	damageadaptive	5
Face Milling	Air-cut	face	16
Face Milling	Active-cut	face 150025	15
Face Milling	Damage	damageface	4
Pocket Machining	Air-cut	pocket	19
Pocket Machining	Active-cut	pocket 150025	19
Pocket Machining	Damage	damagepocket	5
Total			120

Note: Active-cut class labels (suffix 150025) encode the 0.025 in. depth-of-cut parameter from the computer-aided manufacturing configuration; damage class labels carry a damage prefix.

**Table 3:** Parameter Count Breakdown (Active Components for 9-Class Classification).

Component	Parameters	%
Modality Projections (7×)	∼498 K	9.7
Modality Embeddings	1.8 K	<0.1
Cross-Modal Fusion (gates + attention)	∼264 K	5.2
DTAE (2 encoder + 2 decoder layers)	∼3.3 M	64.3
LSTM (2 layers, $d_{\mathrm{model}}\;=\;256$)	∼1.05 M	20.5
Classification Head (256→9)	2.3 K	<0.1
Total (active)	∼5.1 M	100

**Table 4:** Per-Sensor Channel Structure and Encoder Modality Grouping.

Encoder Group	Channel Type	Components	Per Sensor	6 Sensors
Accelerometer	Accelerometer	Ax, Ay, Az	3	18
Gyroscope	Gyroscope	Gx, Gy, Gz	3	18
Magnetometer	Magnetometer	Mx, My, Mz	3	18
Environmental	Pressure	Barometric pressure	1	6
	Temperature	Ambient temperature	1	6
	Proximity	APDS-9960 proximity	1	6
Color	Color	R, G, B, A	4	24
RMS Audio	RMS Audio	PDM microphone RMS	1	6
Electrical	Machine-level (voltage, current, spindle)			8
7 groups			17	110

**Table 5:** Cumulative Feature Ablation Configurations.

#	Configuration	Removed	Features
1	Full	—	110
2	No Proximity	Proximity	104
3	No Proximity + Pressure	Proximity, Pressure	98
4	No Proximity + Pressure + Color	Proximity, Pressure, Color	74
5	Minimal	Proximity, Pressure, Color, Magnetometer	56

**Table 6:** Feature Ablation Results (Window = 64, Stride = 16, 5-Fold Cross-Validation Mean ± Std).

Config	Features	MM-DTAE-LSTM	XGBoost	RF	Logistic	MLP	LSTM
Full	110	93.5 ± 7.2	95.1 ± 4.6	95.6 ± 5.9	92.6 ± 9.4	94.3 ± 8.9	93.3 ± 10.2
No Proximity	104	95.2 ± 8.0	95.5 ± 4.8	95.7 ± 5.5	92.7 ± 9.7	92.8 ± 10.4	92.7 ± 9.3
No Prox + Pres	98	**96.3 ± 4.7**	92.4 ± 6.3	91.1 ± 7.9	92.5 ± 10.0	93.2 ± 9.8	92.5 ± 11.5
No Prox + Pres + Col	74	94.7 ± 8.0	92.4 ± 5.3	91.5 ± 7.6	92.0 ± 9.2	93.4 ± 9.6	91.9 ± 10.1
Minimal	56	92.5 ± 8.3	84.4 ± 9.7	88.0 ± 5.9	85.4 ± 11.7	90.2 ± 11.0	88.7 ± 13.0

**Table 7:** Analysis of Variance Results by Modality Group. Median *F*-statistic and effect size ($\eta^{2}$) across features within each modality. Modalities above the horizontal line were removed during feature ablation.

Modality	Median *F*	Mean *F*	$\eta^{2}$	$N_{\mathrm{feat}}$	$N_{\mathrm{sig}}$
Pressure	2423.2	2067.1	0.994	6	6
Proximity	41.9	44.5	0.749	6	6
Color	17.0	19.8	0.551	24	24
Magnetometer	18.2	167.5	0.568	18	18
RMS Audio	91.4	79.1	0.866	6	6
Gyroscope	11.4	17.9	0.450	18	17
Accelerometer	10.8	26.5	0.438	18	14
Temperature	4.5	4.9	0.246	6	6
Electrical	5.1	5.0	0.271	8	6

**Table 8:** Model Comparison at Best Configuration (No Proximity + Pressure, Window = 64, Stride = 16). Cohen’s *d* is computed from 5 paired fold-level accuracy differences (encoder − baseline); 95% confidence intervals use the *t*-distribution with df = 4.

Model	Accuracy (%)	Macro F1 (%)	Δ Acc	Cohen’s *d*	*p* (Paired *t*)
MM-DTAE-LSTM	**96.3 ± 4.7**	**91.7 ± 8.0**	—	—	—
XGBoost	92.4 ± 6.3	88.7 ± 11.2	+3.8	0.46 [−0.85, 1.76]	0.314
Random Forest	91.1 ± 7.9	86.0 ± 14.6	+5.2	0.74 [−0.66, 2.14]	0.136
Logistic Regression	92.5 ± 10.0	90.7 ± 10.4	+3.8	0.56 [−0.77, 1.90]	0.233
Multilayer Perceptron	93.2 ± 9.8	90.0 ± 12.9	+3.1	0.47 [−0.84, 1.78]	0.299
SimpleLSTM	92.5 ± 11.5	90.8 ± 11.6	+3.8	0.45 [−0.85, 1.75]	0.323

Note on statistical power: With *n* = 5 folds, paired *t*-tests have limited power; *p*-values should be interpreted alongside effect sizes and consistency across folds. Note on configuration: All models evaluated at the encoder’s best feature configuration (98 features). Random Forest peaks at 95.7% with 104 features (Table 6), but diverges sharply from the encoder at reduced feature sets.

**Table 9:** Architectural Component Ablation. All configurations use 98 features, window = 64, stride = 16. Δ columns show the difference from the full model. Full per-class results appear in Table A7 and Table A8 (Appendix D).

Configuration	Mean Acc (%)	Macro F1 (%)	Δ Acc	Δ F1
Full MM-DTAE-LSTM	96.0 ± 6.6	92.1 ± 9.9	—	—
Single Shared Projection	93.8 ± 7.3	90.2 ± 10.0	−2.2	−1.9
No Modality Gates	95.5 ± 6.5	91.5 ± 9.7	−0.5	−0.6
No Cross-Modal Attention	**97.4 ± 3.8**	**93.2 ± 8.1**	+1.4	+1.1
No Denoising Objective	91.6 ± 8.4	84.2 ± 10.5	−4.4	−7.9
No DTAE (Transformer removed)	94.7 ± 6.0	90.4 ± 8.4	−1.3	−1.7
Minimal (Projection → LSTM only)	93.9 ± 8.2	89.7 ± 10.7	−2.1	−2.4

**Table 10:** Model Capacity Ablation. All configurations use the full architectural design with 98 features, window = 64, stride = 16, 5-fold CV. Active parameters exclude auxiliary heads not used during classification inference. *p* vs. 256 from paired *t*-tests (df = 4). One-way ANOVA: F(4,20)=0.046, p=0.996 (accuracy); F(4,20)=0.151, p=0.961 (macro F1).

$d_{\mathrm{model}}$	Active Params	Mean Acc (%)	Macro F1 (%)	*p* vs. 256
32	∼0.1M	97.6 ± 4.3	93.7 ± 7.8	0.071
64	∼0.4M	**98.5 ± 2.7**	**96.4 ± 4.3**	0.488
128	∼1.4M	98.1 ± 3.1	95.7 ± 5.4	0.898
256	∼5.1M	98.0 ± 3.9	94.0 ± 7.7	—
512	∼21.9M	98.4 ± 2.9	96.0 ± 4.9	0.540

**Table 11:** Approximate Sensing Hardware Costs (USD, 2025 Pricing).

Component	Cost
Arduino Nano 33 BLE Sense Lite (×6)	$198
MCC USB-202 data acquisition module (electrical)	$169
Raspberry Pi 4 (data aggregator)	$75
Cabling, breadboards, mounting	∼$50
Total	∼$492

All six sensor boards are required for both the full (110-feature) and minimal (56-feature) configurations; feature ablation removes channel types in software, not physical boards. No controller modification is required.

**Figure A3:**
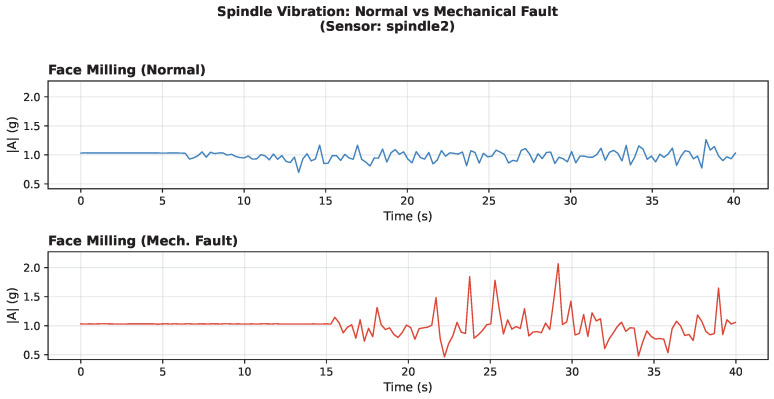
Spindle sensor vibration magnitude comparing normal air-cut face milling (**top**) versus damaged-spindle air-cut face milling (**bottom**). The damage condition shows elevated vibration amplitude due to spindle runout caused by the deliberately removed drive band.

**Figure A4:**
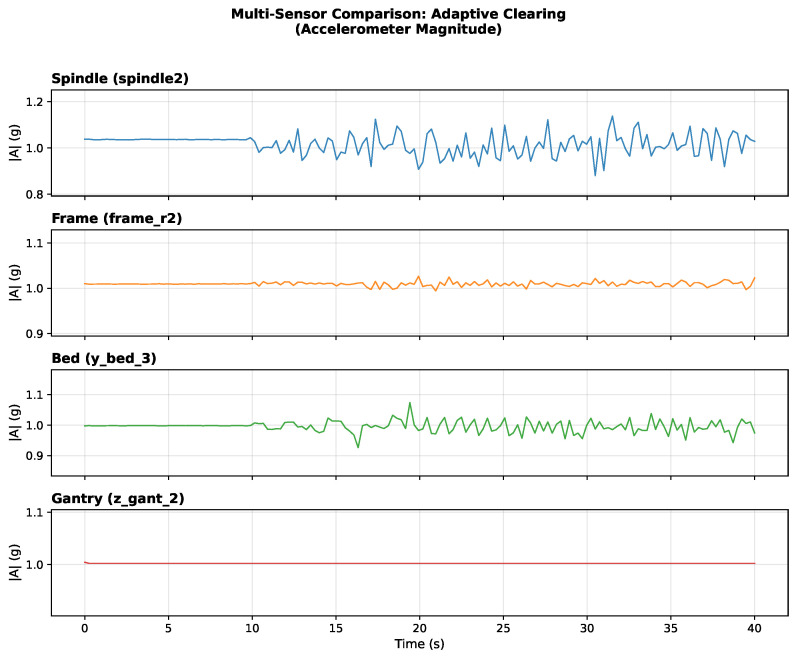
Multi-sensor accelerometer comparison during adaptive clearing (air-cut). Spindle sensors show highest amplitude due to direct proximity to the spindle; bed sensors travel with the workpiece; frame sensors act as structural low-pass filters. Signal strength varies by sensor location, motivating spatially distributed sensor deployment.

**Figure A5:**
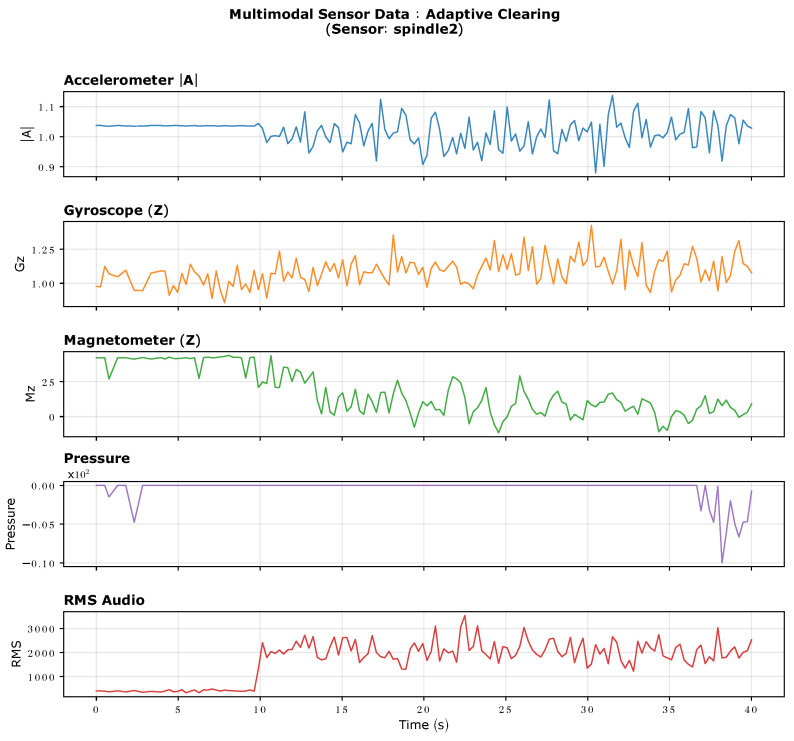
Multimodal sensor data from the spindle sensor during adaptive clearing (air-cut). Each modality captures different physical phenomena: accelerometer magnitude reflects gantry acceleration and structural vibration; gyroscope captures rotational dynamics; magnetometer senses spindle motor magnetic fields; root mean square audio provides acoustic context from airborne emission.

**Figure A7:**
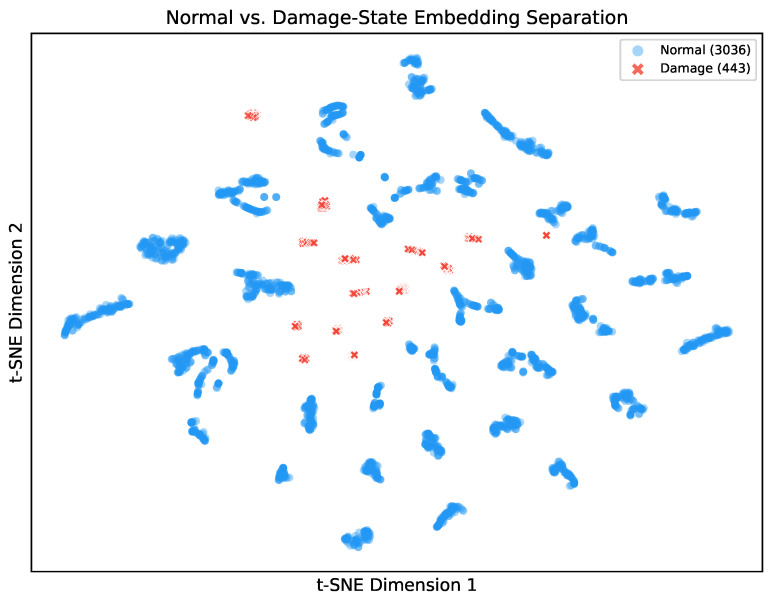
t-Distributed Stochastic Neighbor Embedding visualization of damage versus non-damage embeddings (3479 test samples: 3036 air-cut and active-cut; 443 damage). Damage samples (red markers) are dispersed among non-damage clusters rather than forming a distinct region, confirming that spindle degradation produces subtle signature shifts within each toolpath family rather than a globally separable pattern.

**Figure A11:**
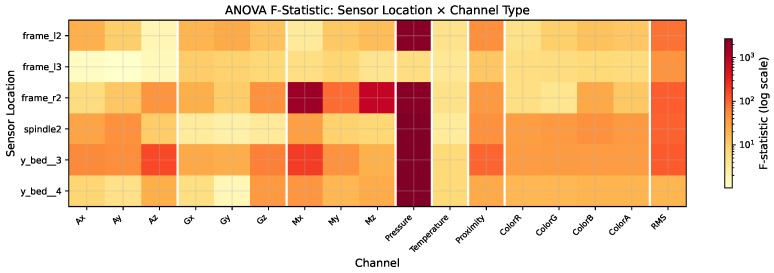
Analysis of variance *F*-statistic heatmap by sensor location and channel type. Pressure channels show uniformly extreme *F* across all sensors (session confound). Magnetometer channels on frame_r2 are outliers (F>800), likely reflecting a consistent magnetic field gradient at that location. Accelerometer and gyroscope channels show moderate, spatially varying *F*-values that reflect genuine location-dependent sensitivity to machine dynamics.

## Data Availability

The sensor datasets and analysis code generated during this study are publicly available at https://github.com/stephenseacuello/gcode_fingerprinting (accessed on 26 February 2026). Source code for the MM-DTAE-LSTM architecture, experiment pipeline, and analysis scripts is available at https://github.com/stephenseacuello/gcode_fingerprinting (accessed on 26 February 2026) under the MIT License.

## Abbreviations

The following abbreviations are used in this manuscript:

ANOVA	Analysis of Variance
AUROC	Area Under the Receiver Operating Characteristic Curve
BLE	Bluetooth Low Energy
CAM	Computer-Aided Manufacturing
CNC	Computer Numerical Control
CPU	Central Processing Unit
CV	Cross-Validation
DTAE	Denoising Transformer Autoencoder
FDM	Fused Deposition Modeling
GPU	Graphics Processing Unit
HSS	High-Speed Steel
IMU	Inertial Measurement Unit
IQR	Interquartile Range
LSTM	Long Short-Term Memory
MCC	Measurement Computing Corporation
MEMS	Micro-Electro-Mechanical Systems
MLP	Multilayer Perceptron
MM-DTAE-LSTM	Multimodal Denoising Temporal Attention Encoder with Long Short-Term Memory
MSE	Mean Squared Error
OT	Operational Technology
PCA	Principal Component Analysis
PDM	Pulse Density Modulation
RMS	Root Mean Square
ROC	Receiver Operating Characteristic
RPM	Revolutions Per Minute
t-SNE	t-Distributed Stochastic Neighbor Embedding
UHMW-PE	Ultra-High Molecular Weight Polyethylene

## Appendix A. Experimental Setup Details

In the following section, we provide detailed information about experimental setup such as the hardware specification, sampling rate, sensor selection, data cleaning, hyperparameter study, and window-stride configuration.

### Appendix A.1. Hardware Specifications

Each Arduino Nano 33 BLE Sense Lite board integrates four sensor components: a nine-axis inertial measurement unit (LSM9DS1: accelerometer, gyroscope, magnetometer), environmental sensors (LPS22HB barometric pressure and temperature, APDS-9960 color and proximity), and a pulse-density modulation microphone (MP34DT05) for root mean square audio computation.

### Appendix A.2. Sampling Configuration

The inertial measurement unit (accelerometer, gyroscope, magnetometer) samples at 119 Hz natively (Nyquist limit 59.5 Hz). Environmental sensors (pressure, temperature, color and proximity) sample at 10 Hz. The on-board microphone computes root mean square audio amplitude at the firmware level before transmission. Motor electrical signals (voltage, current) are captured via MCCDAQ at 1 kHz. All streams are aggregated on a Raspberry Pi 4. Sensor boards communicate via Bluetooth Low Energy wireless links or direct USB serial connections. All streams are then resampled and synchronized to the G-code execution timeline for operation labeling, yielding an effective aligned rate of approximately 4 Hz.

The 400 Hz tooth-passing frequency exceeds the 59.5 Hz inertial measurement unit Nyquist limit, and the ∼4 Hz aligned rate further reduces temporal resolution. However, discriminative information persists. Low-frequency structural vibration modes (1–50 Hz) are excited by gantry motion and, during active cutting, by cutting forces. Acceleration transients occur at toolpath direction changes. Operation-specific trajectory envelopes are preserved at any sampling rate above the motion bandwidth. Classification succeeds because operation type depends on toolpath geometry, which is distinguishable even during air-cutting, rather than on high-frequency cutting dynamics.

### Appendix A.3. Sensor Consistency and Selection

Not all 16 sensors produce consistent data across all files due to intermittent Bluetooth connectivity, USB serial dropouts, and firmware sampling failures. We apply a ≥93% activity threshold (non-null rows across all files) to select reliable sensors:

**Table A1:** Consistently Active Sensors (≥93% Activity Across All Files).

Sensor	Location	Activity (%)	Channels
frame_l2	Frame (left)	>93	17
frame_l3	Frame (left)	>93	17
frame_r2	Frame (right)	>93	17
spindle2	Spindle	>93	17
y_bed_3	Bed	>93	17
y_bed_4	Bed	>93	17
Total sensor features			102
+ Electrical (machine-level)			8
Total features (full configuration)			110

The six selected sensors span three structural groups (frame: 3, spindle: 1, bed: 2), providing spatial diversity across the machine’s primary vibration transmission paths. Sensors at different structural locations observe different projections of the machine’s response to toolpath motion. The spindle sensor captures rotational and axial dynamics close to the cutting zone. Frame and bed sensors capture how vibration propagates through the machine structure. This spatial diversity is a deliberate design choice. A single sensor provides only one projection of machine dynamics, whereas spatially distributed sensors capture complementary information that improves classification robustness.

### Appendix A.4. Data Cleanin

Two cleaning steps reduce the 135 raw files to 120 analysis-ready files:

**(1) Sensor consistency filtering.** Files missing any of the six required sensors (Table A1) are excluded. Of the 15 dropped files, most lack frame_l3 or y_bed_4, the two sensors nearest the 93% activity threshold, due to intermittent connectivity during those recording sessions. This file-level filtering eliminates the need for zero-padding missing sensor columns, which would otherwise allow models to learn from sensor presence or absence rather than from sensor dynamics.

**(2) Trailing NaN truncation.** One file (face150025_001) contained 79% NaN-padded rows caused by a sensor communication dropout partway through the machining operation. These trailing NaN rows were truncated to the last valid sensor reading. Without truncation, forward-fill interpolation would propagate the final valid values across thousands of rows, creating degenerate constant-value windows that corrupt training.

### Appendix A.5. Training Hyperparameters

Table A2 provides detailed hyperparameter configuration that was used during the experiment for reproducibility of the results.

**Table A2:** Training Hyperparameters.

Parameter	Value
Model dimension ($d_{\mathrm{model}}$)	256
LSTM layers	2
Attention heads	4
DTAE layers (encoder + decoder)	2 + 2
DTAE feedforward dimension	$4\times d_{\mathrm{model}}=1024$
Dropout (DTAE, LSTM)	0.2
Dropout (modality projections)	0.1
Modality dropout	0.1
Noise level (σ)	0.05
Mask probability	0.1
Reconstruction weight (λ)	0.1
Label smoothing (ϵ)	0.1
Gradient clipping (max norm)	1.0
Optimizer	AdamW [35] ($\beta_{1}\;=\;0.9$, $\beta_{2}\;=\;0.999$)
Learning rate	$10^{-3}$
Weight decay	0.01
Learning rate schedule	Linear warmup (10 epochs) + Cosine annealing [36]
Cosine restart period	$T_{0}=30$, $T_{\mathrm{mult}}=2$
Max epochs	200
Early stopping patience	40
Batch size	32
Random seed	42

### Appendix A.6. Window and Stride Configurations

Table A3 provides the 10 configurations of window and stride assessed during the experiment.

**Table A3:** Window and Stride Configurations.

Window	Stride	Overlap	Duration (s)	Ratio
16	8	50%	∼4	2.0×
32	8	75%	∼8	4.0×
32	16	50%	∼8	2.0×
64	16	75%	∼16	4.0×
64	32	50%	∼16	2.0×
64	64	0%	∼16	1.0×
128	32	75%	∼32	4.0×
128	64	50%	∼32	2.0×
256	64	75%	∼64	4.0×
256	128	50%	∼64	2.0×

## Appendix B. Supplementary Figures and Tables

This section collects figures and tables that support the Results in Section 4 but were moved out of the main body to preserve reading flow. Training convergence figures appear first (Figure A1 and Figure A2), followed by sensor signal examples (Figure A3, Figure A4 and Figure A5), embedding structure visualizations (Figure A6 and Figure A7), feature and temporal ablation comparisons (Figure A8, Figure A9 and Figure A10), the confound heatmap (Figure A11), and performance tables (Table A4, Table A5 and Table A6).

**Table A4:** Training Time per Fold at Best Configuration (No Proximity and Pressure, Window = 64, Stride = 16). Graphics processing unit times are for a single NVIDIA RTX A6000; central processing unit times are for scikit-learn and XGBoost on the same workstation.

Model	Time (s)	Relative to Encoder
MM-DTAE-LSTM	198 ± 85	1.0× (GPU)
XGBoost	191 ± 15	1.0× (CPU)
Random Forest	1.7 ± 0.2	116× faster
Logistic Regression	10.9 ± 3.0	18× faster
Multilayer Perceptron	672 ± 240	3.4× slower (GPU)
SimpleLSTM	755 ± 234	3.8× slower (GPU)

**Table A5:** Per-Class Accuracy (%, Mean ± Std Across 5 Folds) at Best Encoder Configuration. Damage-class metrics (bottom three rows) are preliminary: with only 4–5 files, some folds contain zero test samples, producing extreme variance that precludes reliable performance estimation.

Class	Files	MM-DTAE-LSTM	XGBoost	RF	MLP	LSTM
adaptive	20	100.0 ± 0.0	93.9 ± 9.3	91.9 ± 11.5	100.0 ± 0.0	100.0 ± 0.0
adaptive 150025	17	99.3 ± 1.1	98.6 ± 3.2	100.0 ± 0.0	99.1 ± 2.0	97.1 ± 6.4
face	16	99.6 ± 1.0	89.4 ± 11.3	93.9 ± 7.9	98.7 ± 1.6	97.0 ± 4.2
face 150025	15	95.6 ± 6.9	93.3 ± 9.6	85.8 ± 23.5	91.5 ± 14.0	85.3 ± 20.4
pocket	19	100.0 ± 0.0	95.0 ± 8.5	91.0 ± 12.4	100.0 ± 0.0	98.7 ± 2.1
pocket 150025	19	93.6 ± 8.5	94.1 ± 11.0	91.6 ± 10.0	80.6 ± 34.4	82.8 ± 33.8
damageadaptive	5	100.0 ± 0.0	100.0 ± 0.0	81.4 ± 41.5	100.0 ± 0.0	100.0 ± 0.0
damageface	4	76.2 ± 43.4	58.1 ± 53.1	69.4 ± 45.1	77.5 ± 43.7	77.5 ± 43.5
damagepocket	5	70.3 ± 44.6	88.0 ± 26.8	80.0 ± 44.7	81.7 ± 40.9	93.1 ± 15.3

**Table A6:** Per-Class F1-Score (%, Mean ± Std Across 5 Folds) at Best Encoder Configuration.

Class	Files	MM-DTAE-LSTM	XGBoost	RF	MLP	LSTM
adaptive	20	100.0 ± 0.0	88.3 ± 13.1	93.0 ± 6.3	100.0 ± 0.0	97.3 ± 3.6
adaptive 150025	17	99.0 ± 1.5	97.2 ± 4.6	100.0 ± 0.0	99.1 ± 2.2	96.8 ± 7.3
face	16	99.0 ± 1.7	92.4 ± 9.5	94.0 ± 5.8	98.7 ± 1.7	98.2 ± 2.6
face 150025	15	94.0 ± 9.3	93.1 ± 6.6	84.8 ± 16.1	91.5 ± 14.8	85.3 ± 20.2
pocket	19	97.8 ± 2.3	91.6 ± 10.7	90.4 ± 9.7	100.0 ± 0.0	93.1 ± 10.4
pocket 150025	19	96.0 ± 5.6	95.2 ± 6.1	90.4 ± 8.4	80.6 ± 38.6	83.4 ± 29.1
damageadaptive	5	100.0 ± 0.0	93.9 ± 12.0	82.7 ± 38.8	100.0 ± 0.0	99.3 ± 1.0
damageface	4	65.8 ± 39.4	56.8 ± 52.0	63.0 ± 41.4	77.5 ± 48.9	72.8 ± 42.4
damagepocket	5	73.6 ± 43.4	89.5 ± 18.2	76.1 ± 43.4	81.7 ± 45.7	91.6 ± 16.5

Note: Logistic Regression is omitted from per-class Table A5 and Table A6 for space; its aggregate performance (92.5% ± 10.0% accuracy, 90.7% ± 10.4% macro F1) appears in Table 8.

## Appendix C. Fingerprint Embedding Details

In addition to the classification head, the model produces a 128-dimensional fingerprint embedding via a dedicated projection head. Attention pooling over the Long Short-Term Memory hidden states yields a fixed-length sequence summary, which is then mapped through a two-layer multilayer perceptron (256→256→128) with Gaussian error linear unit activation and L2 normalization:

(A1) $$f=\frac{{\mathrm{MLP}}_{\mathrm{fp}}\left(\mathrm{AttnPool}\left(H_{\mathrm{lstm}}\right)\right)}{∥{\mathrm{MLP}}_{\mathrm{fp}}\left(\mathrm{AttnPool}\left(H_{\mathrm{lstm}}\right)\right){∥}_{2}}\in R^{128}$$

In the current training configuration, the fingerprint head is not optimized with a dedicated contrastive loss. Instead, it projects the LSTM representations, trained via the combined classification and reconstruction objectives, through an attention-pooled multilayer perceptron. The resulting embeddings inherit their class-discriminative structure from the Long Short-Term Memory hidden states and are used for the visualizations in Figure 4 and Figure A6. The fingerprint head is designed for extension toward contrastive operation-identity learning in future work, where a similarity-based objective could produce tighter intra-class clusters and improve anomaly scoring.

## Appendix D. Architectural Ablation: Full Per-Class Results

Table A7 and Table A8 report per-class F1 and accuracy for all seven architectural configurations. Table A9 and Table A10 report per-fold results. The key pattern visible in the per-class data is that the denoising objective’s contribution is concentrated in the damage classes and the harder within-family condition pairs (face 150025, pocket 150025), while most air-cut and basic active-cut classes are well-classified by all configurations.

**Table A7:** Architectural Ablation—Per-Class F1-Score (%, Mean ± Std Across 5 Folds). Column headers: adapt = adaptive air-cut, adp150 = adaptive active-cut, face = face air-cut, fac150 = face active-cut, pocket = pocket air-cut, poc150 = pocket active-cut, dmgAdp/dmgFac/dmgPoc = damage classes.

Config	Adapt	adp150	Face	fac150	Pocket	poc150	dmgAdp	dmgFac	dmgPoc	Macro F1
Full	99.2 ± 0.8	98.7 ± 2.5	99.0 ± 1.4	94.4 ± 8.5	94.4 ± 10.6	94.0 ± 10.1	99.6 ± 0.7	70.8 ± 38.3	78.6 ± 39.3	92.1 ± 9.9
Single Proj	94.3 ± 9.4	99.9 ± 0.2	98.0 ± 1.4	85.3 ± 17.7	99.3 ± 1.0	90.3 ± 12.5	99.6 ± 0.7	68.4 ± 35.9	76.4 ± 38.4	90.2 ± 10.0
No Gates	99.5 ± 1.1	98.7 ± 2.5	98.7 ± 1.6	92.8 ± 9.2	94.6 ± 10.7	93.4 ± 10.2	100.0 ± 0.0	68.2 ± 36.8	77.2 ± 38.8	91.5 ± 9.7
No CrossAttn	99.1 ± 1.9	99.9 ± 0.2	100.0 ± 0.0	94.7 ± 7.2	97.5 ± 5.0	97.6 ± 4.4	98.4 ± 3.3	72.7 ± 38.3	79.1 ± 39.6	93.2 ± 8.1
No Denoise	96.2 ± 6.1	100.0 ± 0.0	99.5 ± 0.8	83.1 ± 21.5	98.1 ± 2.5	90.5 ± 15.9	92.7 ± 13.8	40.3 ± 33.3	57.6 ± 32.0	84.2 ± 10.5
No DTAE	100.0 ± 0.0	97.9 ± 4.2	100.0 ± 0.0	85.0 ± 20.2	98.9 ± 2.1	94.8 ± 9.4	100.0 ± 0.0	63.1 ± 34.9	73.6 ± 26.2	90.4 ± 8.4
Minimal	100.0 ± 0.0	96.4 ± 7.2	100.0 ± 0.0	90.0 ± 12.5	93.9 ± 11.7	88.5 ± 17.3	100.0 ± 0.0	63.6 ± 34.9	74.9 ± 38.0	89.7 ± 10.7

**Table A8:** Architectural Ablation—Per-Class Accuracy (%, Mean ± Std Across 5 Folds).

Config	Adapt	adp150	Face	fac150	Pocket	poc150	dmgAdp	dmgFac	dmgPoc	Mean Acc
Full	99.2 ± 1.1	99.8 ± 0.4	98.6 ± 2.9	94.7 ± 5.9	99.6 ± 0.9	92.0 ± 16.0	100.0 ± 0.0	76.2 ± 38.3	80.0 ± 40.0	96.0 ± 6.6
Single Proj	99.7 ± 0.6	99.8 ± 0.4	98.7 ± 1.6	81.5 ± 21.5	100.0 ± 0.0	91.1 ± 15.1	99.3 ± 1.4	71.2 ± 37.2	80.0 ± 40.0	93.8 ± 7.3
No Gates	100.0 ± 0.0	100.0 ± 0.0	98.7 ± 2.7	92.2 ± 9.9	100.0 ± 0.0	91.9 ± 16.2	100.0 ± 0.0	71.2 ± 36.8	80.0 ± 40.0	95.5 ± 6.5
No CrossAttn	100.0 ± 0.0	100.0 ± 0.0	100.0 ± 0.0	95.4 ± 6.2	100.0 ± 0.0	96.0 ± 8.1	100.0 ± 0.0	80.0 ± 40.0	78.3 ± 39.3	97.4 ± 3.8
No Denoise	100.0 ± 0.0	100.0 ± 0.0	100.0 ± 0.0	79.7 ± 27.4	100.0 ± 0.0	87.5 ± 19.8	99.3 ± 1.4	54.4 ± 44.6	66.3 ± 42.5	91.6 ± 8.4
No DTAE	100.0 ± 0.0	97.3 ± 5.4	100.0 ± 0.0	80.0 ± 24.5	100.0 ± 0.0	97.3 ± 5.4	100.0 ± 0.0	64.4 ± 38.8	82.9 ± 34.3	94.7 ± 6.0
Minimal	100.0 ± 0.0	100.0 ± 0.0	100.0 ± 0.0	87.7 ± 15.0	100.0 ± 0.0	87.7 ± 24.2	100.0 ± 0.0	65.0 ± 36.1	80.0 ± 40.0	93.9 ± 8.2

**Table A9:** Architectural Ablation—Per-Fold Accuracy (%).

Configuration	Fold 1	Fold 2	Fold 3	Fold 4	Fold 5	Mean ± Std
Full	82.8	99.4	99.9	99.7	98.0	96.0 ± 6.6
Single Proj	79.4	98.0	98.2	98.2	95.2	93.8 ± 7.3
No Gates	82.8	98.7	100.0	99.7	96.2	95.5 ± 6.5
No CrossAttn	90.2	100.0	100.0	99.6	97.5	97.4 ± 3.8
No Denoise	75.1	98.6	96.3	93.1	94.8	91.6 ± 8.4
No DTAE	83.2	98.6	100.0	97.2	94.5	94.7 ± 6.0
Minimal	78.0	99.2	100.0	97.7	94.8	93.9 ± 8.2

**Table A10:** Architectural Ablation—Per-Fold Macro F1 (%).

Configuration	Fold 1	Fold 2	Fold 3	Fold 4	Fold 5	Mean ± Std
Full	74.6	99.3	99.9	99.3	87.3	92.1 ± 9.9
Single Proj	72.6	97.3	98.7	96.6	85.5	90.2 ± 10.0
No Gates	74.7	96.9	100.0	99.3	86.3	91.5 ± 9.7
No CrossAttn	80.6	100.0	100.0	99.0	86.4	93.2 ± 8.1
No Denoise	65.4	96.6	90.9	84.6	83.6	84.2 ± 10.5
No DTAE	78.5	97.6	100.0	92.9	82.9	90.4 ± 8.4
Minimal	70.7	98.0	100.0	94.5	85.2	89.7 ± 10.7

The per-class results in Table A7 and Table A8 reveal where each architectural component contributes. The denoising objective concentrates its benefit on the hardest classes: removing it collapses dmgFac F1 from 70.8% to 40.3% and dmgPoc from 78.6% to 57.6%, while well-represented air-cut classes remain above 96% in all configurations. Cross-modal attention shows no per-class benefit; removing it improves or matches performance on every class. The minimal LSTM-only model matches the full architecture on air-cut and active-cut classes but shows wider variance on damage classes, consistent with the denoising objective providing regularization most valuable for low-data regimes. Per-fold results (Table A9 and Table A10) confirm that fold-to-fold variance is driven primarily by the uneven distribution of damage-class files across folds, not by architectural configuration.

## References

[1] Teti R., Jemielniak K., O’Donnell G., Dornfeld D. (2010). Advanced Monitoring of Machining Operations. CIRP Ann..

[2] Zhong R.Y., Xu X., Klotz E., Newman S.T. (2017). Intelligent Manufacturing in the Context of Industry 4.0: A Review. Engineering.

[3] Stouffer K., Pease M., Tang C., Zimmerman T., Pillitteri V., Lightman S., Hahn A., Saravia S., Sherule A., Thompson M. (2023). NIST Special Publication 800-82 Rev. 3: Guide to Operational Technology (OT) Security.

[4] Serin G., Sener B., Ozbayoglu A.M., Unver H.O. (2020). Review of Tool Condition Monitoring in Machining and Opportunities for Deep Learning. Int. J. Adv. Manuf. Technol..

[5] Zhu K., Wong Y.S., Hong G.S. (2009). Wavelet Analysis of Sensor Signals for Tool Condition Monitoring: A Review and Some New Results. Int. J. Mach. Tools Manuf..

[6] Al Faruque M.A., Chhetri S.R., Wan J., Canedo A. Acoustic Side-Channel Attacks on Additive Manufacturing Systems. Proceedings of the 7th ACM/IEEE International Conference on Cyber-Physical Systems.

[7] Song C., Lin F., Ba Z., Ren K., Zhou C., Xu W. My Smartphone Knows What You Print: Exploring Smartphone-Based Side-Channel Attacks Against 3D Printers. Proceedings of the ACM SIGSAC Conference on Computer and Communications Security.

[8] Chhetri S.R., Canedo A., Al Faruque M.A. KCAD: Kinetic Cyber-Attack Detection Method for Cyber-Physical Additive Manufacturing Systems. Proceedings of the 35th IEEE/ACM International Conference on Computer-Aided Design.

[9] Wang J., Ma Y., Zhang L., Gao R.X., Wu D. (2018). Deep Learning for Smart Manufacturing: Methods and Applications. J. Manuf. Syst..

[10] Zhao R., Yan R., Chen Z., Mao K., Wang P., Gao R.X. (2019). Deep Learning and Its Applications to Machine Health Monitoring. Mech. Syst. Signal Process..

[11] Janssens O., Slavkovikj V., Vervisch B., Stockman K., Loccufier M., Verstockt S., Van de Walle R., Van Hoecke S. (2016). Convolutional Neural Network Based Fault Detection for Rotating Machinery. J. Sound Vib..

[12] Stathatos E., Tzimas E., Benardos P., Vosniakos G.-C. (2024). Convolutional Neural Networks for Raw Signal Classification in CNC Turning Process Monitoring. Sensors.

[13] Bhandari B. (2021). Comparative Study of Popular Deep Learning Models for Machining Roughness Classification Using Sound and Force Signals. Micromachines.

[14] Lahat D., Adali T., Jutten C. (2015). Multimodal Data Fusion: An Overview of Methods, Challenges and Prospects. Proc. IEEE.

[15] Ramachandram D., Taylor G.W. (2017). Deep Multimodal Learning: A Survey on Recent Advances and Trends. IEEE Signal Process. Mag..

[16] Cao L., Li J., Zhang L., Luo S., Li M., Huang X. (2023). Cross-Attention-Based Multi-Sensing Signals Fusion for Penetration State Monitoring During Laser Welding of Aluminum Alloy. Knowl. Based Syst..

[17] Wang H., Wang S., Sun W., Xiang J. (2024). Multi-Sensor Signal Fusion for Tool Wear Condition Monitoring Using Denoising Transformer Auto-Encoder ResNet. J. Manuf. Process..

[18] Tsanousa A., Bektsis E., Kyriakopoulos C., Gómez González A., Leturiondo U., Gialampoukidis I., Karakostas A., Vrochidis S., Kompatsiaris I. (2022). A Review of Multisensor Data Fusion Solutions in Smart Manufacturing: Systems and Trends. Sensors.

[19] Meyes R., Lu M., de Puiseau C.W., Meisen T. (2019). Ablation Studies in Artificial Neural Networks. arXiv.

[20] Jia F., Lei Y., Lin J., Zhou X., Lu N. (2016). Deep Neural Networks: A Promising Tool for Fault Characteristic Mining and Intelligent Diagnosis of Rotating Machinery with Massive Data. Mech. Syst. Signal Process..

[21] Hochreiter S., Schmidhuber J. (1997). Long Short-Term Memory. Neural Comput..

[22] Sutskever I., Vinyals O., Le Q.V. Sequence to Sequence Learning with Neural Networks. Proceedings of the Advances in Neural Information Processing Systems.

[23] Bahdanau D., Cho K., Bengio Y. Neural Machine Translation by Jointly Learning to Align and Translate. Proceedings of the 3rd International Conference on Learning Representations.

[24] Vaswani A., Shazeer N., Parmar N., Uszkoreit J., Jones L., Gomez A.N., Kaiser L., Polosukhin I. Attention Is All You Need. Proceedings of the Advances in Neural Information Processing Systems.

[25] Wen Q., Zhou T., Zhang C., Chen W., Ma Z., Yan J., Sun L. Transformers in Time Series: A Survey. Proceedings of the Thirty-Second International Joint Conference on Artificial Intelligence.

[26] Malhotra P., Vig L., Shroff G., Agarwal P. Long Short Term Memory Networks for Anomaly Detection in Time Series. Proceedings of the 23rd European Symposium on Artificial Neural Networks.

[27] Vincent P., Larochelle H., Lajoie I., Bengio Y., Manzagol P.-A. (2010). Stacked Denoising Autoencoders: Learning Useful Representations in a Deep Network with a Local Denoising Criterion. J. Mach. Learn. Res..

[28] Coelho P.J., Athar A., Mozumder M.A.I., Ali S., Kim H.-C. (2024). Deep Learning-Based Anomaly Detection Using One-Dimensional Convolutional Neural Networks (1D CNN) in Machine Centers (MCT) and Computer Numerical Control (CNC) Machines. PeerJ Comput. Sci..

[29] Kimmell J.C., Orlyanchik V., Sturm L., Dawson J., Taylor C. (2025). Detecting Control Injection Attacks Using Energy Data Anomalies in Computer Numerical Control Machining. Critical Infrastructure Protection XVIII; IFIP Advances in Information and Communication Technology.

[30] Jamarani A., Tu Y., Hei X. (2025). Practitioner Paper: Decoding Intellectual Property: Acoustic and Magnetic Side-Channel Attack on a 3D Printer. Security and Privacy in Cyber-Physical Systems and Smart Vehicles. SmartSP 2024; Lecture Notes of the Institute for Computer Sciences, Social Informatics and Telecommunications Engineering.

[31] Chattopadhyay T., Ceschin F., Garza M.E., Zyunkin D., Chhotaray A., Stebner A.P., Zonouz S., Beyah R. One Video to Steal Them All: 3D-Printing IP Theft through Optical Side-Channels. Proceedings of the 2025 ACM SIGSAC Conference on Computer and Communications Security.

[32] Ba J.L., Kiros J.R., Hinton G.E. (2016). Layer Normalization. arXiv.

[33] Srivastava N., Hinton G., Krizhevsky A., Sutskever I., Salakhutdinov R. (2014). Dropout: A Simple Way to Prevent Neural Networks from Overfitting. J. Mach. Learn. Res..

[34] Szegedy C., Vanhoucke V., Ioffe S., Shlens J., Wojna Z. Rethinking the Inception Architecture for Computer Vision. Proceedings of the 2016 IEEE Conference on Computer Vision and Pattern Recognition.

[35] Loshchilov I., Hutter F. Decoupled Weight Decay Regularization. Proceedings of the 7th International Conference on Learning Representations.

[36] Loshchilov I., Hutter F. SGDR: Stochastic Gradient Descent with Warm Restarts. Proceedings of the 5th International Conference on Learning Representations.

[37] Pedregosa F., Varoquaux G., Gramfort A., Michel V., Thirion B., Grisel O., Blondel M., Prettenhofer P., Weiss R., Dubourg V. (2011). Scikit-learn: Machine Learning in Python. J. Mach. Learn. Res..

[38] Chen T., Guestrin C. XGBoost: A Scalable Tree Boosting System. Proceedings of the 22nd ACM SIGKDD International Conference on Knowledge Discovery and Data Mining.

[39] Breiman L. (2001). Random Forests. Mach. Learn..

[40] Virtanen P., Gommers R., Oliphant T.E., Haberland M., Reddy T., Cournapeau D., Burovski E., Peterson P., Weckesser W., Bright J. (2020). SciPy 1.0: Fundamental Algorithms for Scientific Computing in Python. Nat. Methods.

[41] Jolliffe I.T., Cadima J. (2016). Principal Component Analysis: A Review and Recent Developments. Philos. Trans. R. Soc. A.

